# Developing the Inpatient Mental Health Pharmaceutical Assessment and Care Tool (IMPACT) for use by UK mental health pharmacy teams—a modified Delphi study

**DOI:** 10.1002/bcp.70083

**Published:** 2025-05-23

**Authors:** Fatima Q. Alshaikhmubarak, Richard N. Keers, Petra Brown, Penny J. Lewis

**Affiliations:** ^1^ Division of Pharmacy and Optometry The University of Manchester Manchester UK; ^2^ NIHR Greater Manchester Patient Safety Research Collaboration Manchester UK; ^3^ Optimising Outcomes with Medicines (OptiMed) Research Unit, Pennine Care NHS Foundation Trust Manchester UK; ^4^ Manchester University NHS Foundation Trust Manchester UK

**Keywords:** acuity, clinical pharmacy, inpatient, mental health, patient prioritization, psychiatry

## Abstract

**Aims:**

To develop an evidence‐ and consensus‐based patient prioritization tool for use by UK mental health inpatient pharmacy teams.

**Methods:**

A modified‐Delphi technique was used to obtain experts' agreement on the content, design and practical use of the patient prioritization tool. Two sequential Delphi questionnaires were prepared based on published evidence concerning risk factors for drug‐related problems in mental health hospitals and current prioritization practices used by UK mental health inpatient pharmacy teams. Questionnaires were discussed and agreed upon with a group of 5 stakeholders including 3 mental health pharmacy experts and 2 patient representatives. Pharmacy professionals, psychiatrists and academics were recruited through a previous study and professional networks. Agreement was achieved if ≥75 or ≥85% of the panel rated 6–7 or 5–7 in the Likert scale respectively.

**Results:**

In Delphi 1 questionnaire, 29 experts agreed to include 82 risk indicators in the tool and to categorize patients into 3 risk groups using a traffic light system (red = high‐risk, amber = medium‐risk, green = low‐risk). In Delphi 2 questionnaire, 30 experts agreed on 13 statements guiding practical use of the tool and the preferred frequency of review was *every 1–2 day* for the high‐risk group, *every 2–4 days* for the medium‐risk group and *once every working week* for the low‐risk group.

**Conclusion:**

This study developed the Inpatient Mental Health Pharmaceutical Assessment and Care Tool (IMPACT) for use by UK mental health pharmacy teams. Feasibility and acceptability testing should be carried out to refine the tool and support preparations for formal evaluation of its impact on patient care and service delivery.

**What is already known about this subject**

- Prioritizing inpatients for pharmacy services may improve patient care and enhance pharmacy service delivery.
- There is a need for a standardized patient prioritization tool for use by mental health inpatient pharmacy teams in the UK.

**What this study adds**

- This study developed an evidence‐ and consensus‐based patient prioritization tool for use by UK mental health inpatient pharmacy teams.
- The Inpatient Mental Health Pharmaceutical Assessment and Care Tool might significantly improve mental health pharmacy service delivery following feasibility and acceptability testing.

## 1 INTRODUCTION

The number of patients accessing secondary mental health services in the UK is increasing, with the most recent statistics reporting over 2 million patients accessing secondary mental health services in 2020–2021 alone.^1^, ^2^ With medications playing a major role in the treatment of mental illnesses,^3^ it is not surprising that drug‐related problems (e.g. medication errors and adverse drug events) are a major challenge in this setting, especially with frequent use of high‐risk medications such as lithium^4^, ^5^ and clozapine.^6^ A common definition for a drug‐related problem is “an event or circumstance involving drug therapy that actually or potentially interferes with desired health outcomes”.^7^ Drug‐related problems are common in mental health units with a recent study from England reporting a rate of 2.6 adverse drug events per 1000 patient days.^8^

The vital role of clinical pharmacists in detecting and preventing drug‐related problems within inpatient mental health wards has been demonstrated in published studies.^9^, ^10^ However, the increased service demand prompted by the growing numbers of patients accessing secondary mental health services^2^ and the expanding roles and responsibilities of pharmacists^11^ (e.g. leading clozapine clinics, prescribing independently and delivering outpatient nicotine replacement therapy services^12^) and pharmacy technicians (e.g. technician accuracy checking)^13^ may mean spending less time on inpatient ward duties. This may adversely impact on the ability of pharmacy teams to deliver the desired level of care to all hospital inpatients. Other challenges facing mental health services include limited NHS funding and staff shortages.^14^, ^15^, ^16^ This creates the need for an intervention to support mental health pharmacy teams in operating efficiently to meet demand and provide quality care despite constrained resources.

A recent study aimed to prioritize medication safety research topics using expert consensus and reported that developing approaches to prioritize patients based on their predicted risk was a high priority area.^17^ Such approaches were developed and shown to be effective in enhancing pharmacy service delivery and improving patient outcomes in acute hospitals in the UK,^18^ and internationally^19^ where increasing workload and limited resources are also recognized.^20^ Additionally, our research exploring inpatient pharmacy prioritization approaches in UK mental health organizations found that 21 (*n =* 21/55, 38%) were using prioritization approaches and others were keen to use them in the future due to their promising value. However, due to pharmacists' busy schedules and organizations' limited resources, most of the currently used approaches were relatively simple (involving a few instructions in the standard operating procedures or a list of high‐risk indicators) and developed informally based on existing expertise.^21^ This means that it is important to develop a prioritization tool grounded in both expertise and evidence of risk factors for drug‐related problems in mental health to guide mental health pharmacy teams in prioritizing patients in the most effective way.

Our international systematic review of 22 published studies explored risk factors for drug‐related problems in mental health units, finding a limited number of studies that could not be meta‐analysed due to heterogeneity.^22^ The review, however, identified several risk factors from existing studies and highlighted important considerations for future studies exploring risk factors for drug‐related problems. The lack of evidence in the literature moves us toward a different methodology to attain a rigorously established list of indicators, the Delphi technique. The Delphi consensus method is ideal for such situations where robust evidence is scarce and is also useful for improving decision making.^23^ It was also used to develop patient prioritization tools in pharmacy^24^ and for other healthcare services.^25^, ^26^ A systematic review of patient prioritization tools in healthcare recommended conducting a systematic review followed by a consensus method (e.g. Delphi) to develop rigorous tools.^26^ It also recommended involving stakeholders in the tool development and adapting the tool to the context to ensure its acceptability and practicality.^26^ Therefore, with support from stakeholders, available evidence was combined with expert opinion through a modified Delphi technique to decide upon high‐risk indicators to be included in the tool and to agree on the use and application of the patient prioritization tool.

## 2 AIM

The aim of this study was to develop an evidence‐ and consensus‐based patient prioritization tool for use by mental health inpatient pharmacy teams.

## 3 METHODS

### 3.1 Tool development process

**FIGURE 1:**
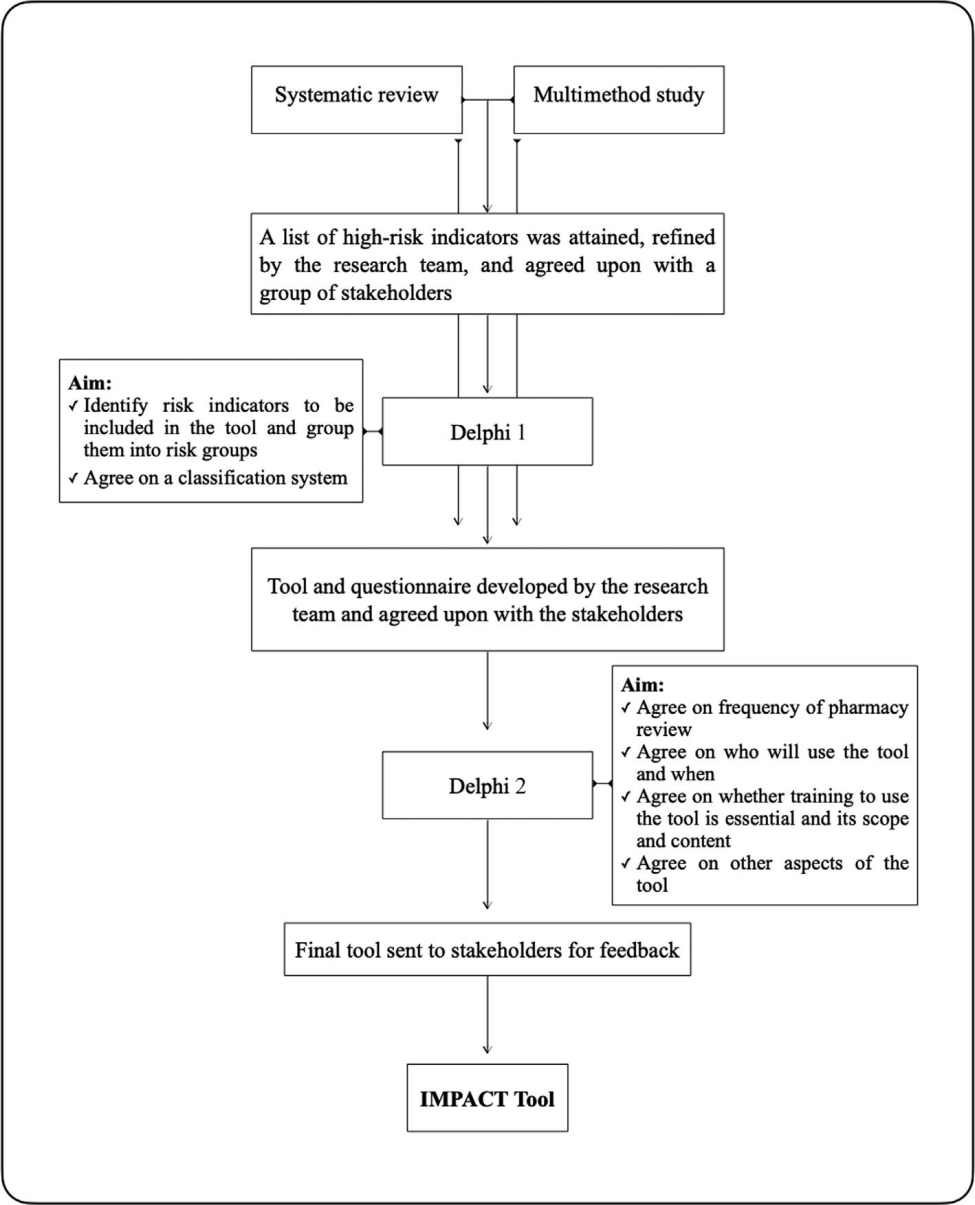
The IMPACT tool development pathway.

Two Delphi studies were performed to develop the Inpatient Mental Health Pharmaceutical Assessment and Care (IMPACT) tool. Delphi study 1 aimed to reach a consensus on which risk indicators for drug‐related problems would be included in the prioritization tool, group them into risk groups and explore agreement on a classification system. Delphi study 2 aimed to reach consensus on how and when the tool should be used, as well as any further additions or modifications. An outline of the tool development process is shown in Figure 1. Both Delphi questionnaires were developed using Qualtrics^27^ survey software and emailed to participants with details and instructions for completing the questionnaire. Participants were offered a £25 shopping voucher to thank them for their time after the second round of each Delphi questionnaire.

### 3.2 Delphi study 1

#### 3.2.1 Delphi 1: questionnaire components

A list of risk indicators was obtained from our previous systematic review^22^ and from our multimethod study exploring existing prioritization approaches in UK mental health units.^21^ The multimethod study included semistructured interviews with senior pharmacy team members who used prioritization approaches and document analysis of their existing prioritization documents.^21^ Risk indicators for drug‐related problems extracted from these studies were collated by FQA into 4 groups to facilitate their handling (high‐risk medicines, patient‐related, drug‐related and hospital‐related indicators). These were then shared and discussed with the research team to ensure clarity and coherent presentation.

#### 3.2.2 Delphi 1: stakeholder group meeting

The risk indicators list and the initial questionnaire design were then discussed by the research team with a group of 5 stakeholders. The stakeholders consisted of 2 patient representatives with prior inpatient experience in a mental health unit and 3 mental health pharmacy team members with inpatient experience (1 deputy chief pharmacist, 1 advanced clinical pharmacist and 1 pharmacy technician) from 3 different mental health trusts in northern England to capture varied experiences. The stakeholder group members were offered a shopping voucher of £50 for their time. The 2.5 h meeting was held during June 2023 where each group of risk indicators and the questionnaire design were discussed with agreements made around exclusion of some indicators. Due to the length of the risk indicators list, it was not possible to discuss each risk indicator individually; however, in many cases, the group agreed on approaches to manage groups of indicators.

Guiding principles for inclusion and exclusion of risk indicators (Appendix 1) were then written by the research team based on the stakeholder group discussion. These guiding principles, along with written comments and agreements made in the stakeholders group meeting, were used by the research team to make final decisions on what risk indicators for drug‐related problems were included in the Delphi questionnaire and the overall questionnaire design. A summary of risk indicators that were removed, modified, or added is available in Appendix 2. The draft questionnaire was finalized and piloted with 4 pharmacists of different backgrounds before commencing the first Delphi round.

#### 3.2.3 Delphi 1: questionnaire design

A fully labelled 7‐point Likert scale was used to reduce misresponse according to a suggested framework for selecting response scales^28^ and, as 7 is an odd number, this ensured the presence of a neutral point.^29^ The statements included risk indicators as well as suggested classification systems for risk categories (e.g. red, amber and green). Panellists were asked to rate each indicator in terms of its importance for inclusion in the prioritization tool (very important, important, somewhat important, neutral, somewhat unimportant, not important, extremely unimportant) and the risk category a patient with this indicator should fall within (extremely high risk, high risk, somewhat high risk, neutral, somewhat low risk, low risk, extremely low risk). The Likert scale included an *outside my expertise* option to ensure the middle point, neutral, did not serve multiple functions^30^ avoiding a previously reported problem of panel members choosing neutral when they had no opinion.^31^ Moreover, panellists were given the opportunity to justify their choice after rating each risk indicator and to suggest additional risk indicators at the end of the questionnaire. As for the indicator classification systems, panellists were asked to rate their agreement with each proposed system (strongly agree, agree, somewhat agree, neutral, somewhat disagree, disagree, strongly disagree) and to suggest other classification systems if they wished.

#### 3.2.4 Delphi 1: sample selection and recruitment

We invited 42 experts from different backgrounds: mental health pharmacists with varied experience; inpatient psychiatrists; and academics with knowledge and expertise in medication safety in mental health. Pharmacy experts were required to have a minimum of 3 years recent experience within mental health inpatient care.

Experts were identified through our previous study^21^ (if they consented to be contacted about further studies) and the research team's professional network. A mix of purposive sampling and snowball sampling were used and recruitment started in August 2023. Delphi 1 commenced towards the end of September 2023.

#### 3.2.5 Delphi 1: definition of consensus

An indicator was included in the tool if ≥75% of the panel rated it as important or very important (Likert ratings: 6–7) or ≥85% rated it as somewhat important, important or very important (Likert ratings: 5–7). Included indicators were considered high risk if ≥75% of panellists rated them as high risk or extremely high risk (Likert ratings: 6–7), moderate risk if ≥75% rated them as somewhat high risk, neutral or somewhat low risk (Likert ratings: 3–5), and low risk if ≥75% rated them as low risk or extremely low risk (Likert ratings: 1–2).

#### 3.2.6 Delphi 1: round 1

The first questionnaire was expected to take around 45 min to complete based on the pilot and the panel were given 4 weeks to complete it. Two reminders were sent to the panel members during this 4‐week period and nonresponders were given an extension of 1 week to take into account their busy schedules.

#### 3.2.7 Delphi 1: controlled feedback

Once the first round was completed, responses were analysed to prepare controlled feedback for the panellists. The following analysis approach was undertaken:

1. The free text‐box comments were analysed thematically while trying to present the summaries as concisely and close to the original statements as possible, in an aim to minimize researcher bias and avoid high dropout rates.
2. Descriptive statistics were calculated using Microsoft Excel.
3. Participants' comments were used to modify or add new risk indicators.

**FIGURE 2:**
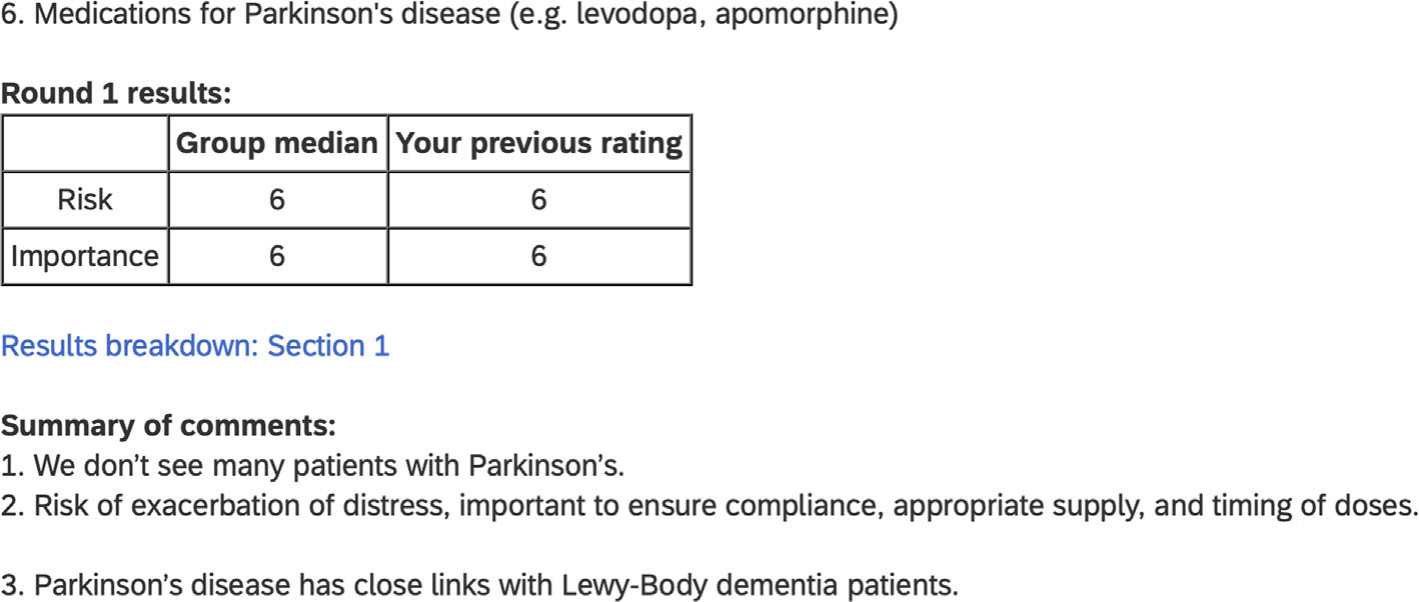
Example feedback given in the second round of Delphi study 1.

The feedback was then presented to participants along with their previous ratings, and included narrative summaries, medians of ratings for each risk indicator and a link to the breakdown of results showing how many panel members selected each rating score (see example in Figure 2). Additionally, the sentence “*Newly added indicator” was added below each new risk indicator (*n =* 14) and “Modified from: *original risk indicator*” was added below each modified risk indicator (*n =* 8).

#### 3.2.8 Delphi 1: round 2

A second round of the questionnaire was sent to panellists along with controlled feedback, to allow them to reconsider their initial responses. All risk indicators from round 1 were included in the second round (8 were modified), and 14 additional risk indicators were added based on panellists' comments and suggestions (see Appendix 3). As the options for possible classification systems received similar panel ratings in round 1, this question was modified to a ranking question, where panel members were asked to rank the classification systems from the most preferred to the least preferred.

### 3.3 Delphi 2

#### 3.3.1 Delphi 2: questionnaire components

Based on findings from Delphi study 1 and the multimethod study,^21^ a draft for the IMPACT tool and a draft questionnaire for Delphi study 2 were developed by FQA. Delphi 2 questionnaire included statements about the use and application of the tool such as the frequency and time of pharmacy review, incorporating clinical judgement and training.

#### 3.3.2 Delphi 2: stakeholder group meeting

The tool and questionnaire were discussed and refined with the research team, before being discussed at a second stakeholder group meeting held via MS Teams during February 2024. This second meeting included a discussion of some Delphi study 1 risk indicators, the Delphi study 2 questionnaire and draft of the patient prioritization tool. It was agreed to exclude 1 risk indicator and modify another. It was also agreed that when a risk indicator did not meet the high‐risk nor the medium‐risk criteria, but the majority rated it as medium or high risk, it would be considered medium risk. The draft of the tool was presented and feedback was sought regarding its structure, pertinence and feasibility. The meeting lasted 2 hours with minor changes suggested by stakeholders, who were offered a £50 shopping voucher for their time. The questionnaire was finalized by the research team after the meeting and piloted with 2 pharmacists, 1 of whom worked in mental health, before commencing Delphi study 2.

#### 3.3.3 Delphi 2: questionnaire design and definition of consensus

Similar to Delphi 1, a 7‐point Likert scale was used to rate the statements (strongly agree, agree, somewhat agree, neutral, somewhat disagree, disagree, strongly disagree) and controlled feedback was provided to the panel after the first round. A statement was considered important if ≥75% of the panel rated it as agree and strongly agree (Likert rating: 6–7) or ≥85% rated it as somewhat agree, agree and strongly agree (Likert ratings: 5–7).

#### 3.3.4 Delphi 2: recruitment

The same panel members of Delphi study 1 were invited to take part in Delphi study 2 except for academics and psychiatrists. As Delphi study 2 was mainly focused on practical aspects of using the tool, familiarity with hospital pharmacy work and daily activities was deemed necessary. Hence, pharmacy technicians and additional senior pharmacists were invited to take part in Delphi study 2 using the same recruitment process as Delphi study 1. Delphi study 2 commenced in March 2024.

#### 3.3.5 Delphi 2: round 1

The questionnaire was emailed to panel members along with instructions for completion. Completion was expected to take around 20 min and participants were given 2 weeks to complete it and 2 reminders were sent.

#### 3.3.6 Delphi 2: controlled feedback

The same analysis approach used in Delphi study 1 was used for Delphi study 2.

**FIGURE 3:**
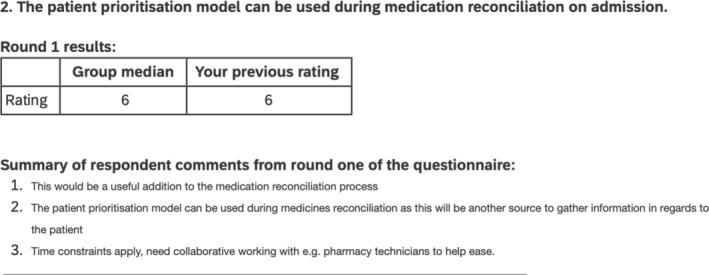
Example feedback given in the second round of Delphi study 2.

The feedback presented to participants along with their previous ratings included narrative summaries, medians of ratings for each risk indicator (see example in Figure 3). One additional statement was added for round 2, and 2 statements were modified (see Appendix 4).

#### 3.3.7 Delphi 2: round 2

In this round, we added the summary of both quantitative and qualitative responses to the questionnaire along with the additional statement. In addition, we added a ranking question for participants to rank their preference for the frequency of review of each risk category. This was added as there was no agreement on any frequency in round 1 and we wanted to ensure that the choice was based on participants' opinions. Last, 1 open‐ended question was added at the end of the questionnaire to explore participants' preferred format for the tool. The amendments made to the tool based on participants' comments can be seen in Appendix 4.

### 3.4 Data analysis

Quantitative results were analysed using Microsoft Excel to calculate the descriptive statistics and open‐ended questions were analysed thematically.

## 4 RESULTS

### 4.1 Delphi study 1

A total of 42 experts agreed to participate in this questionnaire with 36 (85.7%) completing the first round and 29 (80.6%) completing the second round. Panel members who completed round 1 had experience ranging from 3 to over 20 years and their professions are described in Table 1. The majority had their recent experience in England (80.6%, *n =* 29/36), followed by Scotland (13.9%, *n =* 5/36), with 2 in Wales.

**TABLE 1:** Panel members' professions.

Profession	Delphi study 1		Delphi study 2	
	Round 1	Round 2	Round 1	Round 2
**Chief pharmacists**	5	5	5	4
**Senior pharmacists**	22	16	17	16
**Clinical pharmacists**	4	4	3	3
**Chief pharmacy technicians**			1	1
**Senior pharmacy technicians**			5	5
**Pharmacy technicians**			1	1
**Psychiatrists**	3	2		
**Academics**	2	2		

A list of 460 risk indicators was obtained from the systematic review (*n =* 48) and the multimethod study (*n =* 415) with 3 risk indicators reported in both. These risk indicators were then refined and condensed by the research team and stakeholders to 109 risk indicators. In the first round, 43.1% (47/109) met our inclusion criteria for consensus (Table 2 and Appendix 5). In the second round, after reviewing the panel feedback, there was more agreement between the experts and 66.7% (82/123) risk indicators met our inclusion criteria (Table 2 and Appendix 5).

**TABLE 2:** Results of both rounds of Delphi study 1.

	Risk indicators	Risk^#^						Importance^##^					
		Round 1			Round 2			Round 1			Round 2		
		Median	3–5%	6–7%	Median	3–5%	6–7%	Median	5–7%	6–7%	Median	5–7%	6–7%
1	Antipsychotics (e.g. risperidone, haloperidol)	6	41.7	55.6	6	31.0	69.0	6	94.4	72.2	6	100.0	75.9
2	Anticonvulsants (e.g. topiramate, levetriacetam). Round 2: Anticonvulsants (e.g. topiramate, levetriacetam) for epilepsy	6	25.0	75.0	6	17.2	82.8	6	86.1	66.7	6	100.0	75.9
3	Strong opioids (e.g. methadone, fentanyl)	6.5	13.9	86.1	6	6.9	93.1	6	94.4	77.8	6	100.0	82.8
4	Clozapine	7	2.8	97.2	7	0.0	100.0	7	100.0	100.0	7	100.0	100.0
5	Lithium	7	2.8	97.2	7	0.0	100.0	7	100.0	97.2	7	100.0	100.0
6	Insulin	7	5.7	94.3	7	0.0	100.0	7	100.0	88.6	7	100.0	100.0
7	Valproate	6	16.7	83.3	6	6.9	93.1	6	91.7	86.1	6	100.0	89.7
8	Missed mental health medications	5.5	50.0	50.0	5	51.7	48.3	6	100.0	66.7	6	100.0	65.5
9	CKD Stage > 3a (eGFR of 45 to 59 mL/min)	6	30.6	69.4	6	27.6	72.4	6	86.1	61.1	6	100.0	75.9
10	CKD Stage > 3b (eGFR of 30 to 44 mL/min)	6	16.7	83.3	6	13.8	86.2	6	85.7	77.1	6	100.0	86.2
11	CKD Stage > 4 (eGFR of 15 to 29 mL/min)	7	0.0	100.0	7	0.0	100.0	7	88.2	88.2	7	100.0	100.0
12	CKD Stage > 5 (eGFR below 15 mL/min)	7	0.0	100.0	7	0.0	100.0	7	87.9	87.9	7	100.0	100.0
13	Patients with new swallowing difficulties	6	20.0	80.0	6	17.9	82.1	6	85.7	60.0	6	100.0	75.0
14	Formulation review required e.g. NG, PEG, JEJ	6	25.0	75.0	6	11.5	88.5	6	75.0	59.4	6	100.0	80.8
15	Patients with Behavioural and Psychological Symptoms of Dementia when not on a dementia ward	6	32.4	67.6	6	29.6	70.4	5	81.8	48.5	5	100.0	44.4
16	Patients receiving covert medications	6	36.1	63.9	6	24.1	75.9	6	83.3	58.3	6	100.0	75.9
17	Patients regularly spitting out or refusing medication	6	36.1	61.1	6	27.6	69.0	6	94.4	61.1	6	100.0	79.3
18	Patients with high creatine kinase	6	37.1	62.9	6	28.6	71.4	6	82.9	51.4	6	100.0	64.3
19	Toxic clozapine serum levels	7	0.0	100.0	7	0.0	100.0	7	97.2	94.4	7	100.0	100.0
20	Toxic lithium blood levels	7	0.0	100.0	7	0.0	100.0	7	97.2	94.4	7	100.0	100.0
21	QTc results outside reference range	6	11.1	88.9	6	6.9	93.1	6	97.2	80.6	6	100.0	93.1
22	Patients prescribed medicines as part of a clinical trial	6	30.3	69.7	6	16.0	84.0	6	81.8	63.6	6	100.0	80.0
23	Patients with unverified newly started medication	6	41.7	58.3	6	27.6	72.4	6	88.9	63.9	6	100.0	75.9
24	Patients prescribed unlicensed medicines	5	52.8	44.4	5	58.6	41.4	5	80.6	41.7	5	100.0	34.5
25	Sudden/abrupt cessation of medication	6	36.1	63.9	6	24.1	75.9	6	91.7	58.3	6	100.0	75.9
26	Depot antipsychotics	6	47.2	52.8	6	31.0	69.0	6	88.9	58.3	6	96.6	75.9
27	Zuclopenthixol acetate or zuclopenthixol acuphase	6.5	8.3	91.7	7	0.0	100.0	7	91.7	86.1	7	96.6	96.6
28	Presence of a significant ^a^ adverse drug reaction	7	11.1	88.9	7	3.4	96.6	7	97.2	86.1	7	96.6	96.6
29	Significant ^b^ drug interaction	6	5.6	94.4	6	6.9	93.1	6	100.0	86.1	6	96.6	86.2
30	More than 1 regular antipsychotic prescribed	6	22.2	77.8	6	13.8	86.2	6	97.2	88.9	6	96.6	89.7
31	More than 1 hypnotic prescribed	6	44.4	52.8	6	31.0	69.0	6	88.9	61.1	6	96.6	58.6
32	Patient with dementia or cognitive impairment prescribed 1 or more antimuscarinics				6	17.2	82.8				6	96.6	75.9
33	Missed doses	5.5	50.0	50.0	5	51.7	48.3	6	94.4	52.8	5	96.6	44.8
34	Missed doses of high‐risk medications				6	3.4	96.6				6	96.6	93.1
35	Missed doses of high‐risk mental health medications				6	6.9	93.1				6	96.6	93.1
36	High‐dose antipsychotic therapy (above 100% BNF maximum) prescribed	6	2.8	97.2	6	6.9	93.1	6	97.2	86.1	6	96.6	93.1
37	Any single drug above BNF limits (unless planned detoxification)	6	19.4	80.6	6	41.4	58.6	6	91.7	66.7	6	96.6	65.5
38	Female of childbearing potential prescribed sodium valproate Round 2: Female of childbearing potential prescribed teratogenic medicines such as sodium valproate	7	0.0	100.0	7	0.0	100.0	7	97.2	97.2	7	96.6	96.6
39	Patient requires intramuscular rapid‐tranquillisation administration	6	11.1	88.9	6	10.3	89.7	6.5	97.2	86.1	6	96.6	86.2
40	Acute renal impairment	6.5	8.3	91.7	6	3.4	96.6	6	88.9	83.3	6	96.6	89.7
41	Chronic hepatic impairment	6	19.4	80.6	6	20.7	79.3	6	80.6	66.7	6	96.6	72.4
42	Patients receiving electroconvulsive therapy ^c^	6	27.8	72.2	6	20.7	79.3	6	88.9	61.1	6	96.6	79.3
43	Patients with nonadherence				6	37.9	62.1				6	96.6	65.5
44	Electrolytes levels outside reference range	6	44.4	55.6	6	37.9	62.1	5	88.9	44.4	5	96.6	34.5
45	Patient not reviewed within the past 7 days (acute) or fortnight (rehab) by a pharmacist	5	55.6	44.4	5	58.6	41.4	6	83.3	63.9	6	96.6	62.1
46	Polypharmacy ≥ 10 regular medications	6	13.9	86.1	6	10.3	89.7	6	91.7	77.8	6	96.6	79.3
47	New T2/T3 under the Mental Health Act	5	58.3	36.1	5	75.9	20.7	6	91.7	55.6	5	96.6	48.3
48	Patients with swallowing difficulties/Nil by mouth	6	23.5	73.5	6	7.1	89.3	6	85.3	67.6	6	96.4	78.6
49	Acute hepatic impairment (liver function tests >3× upper limit of normal)	6	14.7	85.3	6	3.7	96.3	6	82.4	67.6	6	96.3	92.6
50	Medications for Parkinson's disease (e.g. levodopa, apomorphine)	6	25.0	75.0	6	17.2	82.8	6	83.3	52.8	6	93.1	69.0
51	Anticoagulants Round 2: Prescribed direct oral anticoagulant medication	6	27.8	72.2	6	13.8	86.2	6	86.1	52.8	6	93.1	79.3
52	Low sodium levels in a patient taking 1 or more antidepressants				6	37.9	62.1				6	93.1	65.5
53	Increase of a regular psychotropic within 7 days of the last increase (unless as part of a dose titration regimen)	5	52.8	47.2	5	58.6	41.4	5.5	88.9	50.0	5	93.1	41.4
54	Patient requires rapid tranquillization Round 2: Patient requires oral when required psychotropic for agitation	6	33.3	66.7	6	37.9	62.1	6	94.4	63.9	6	93.1	62.1
55	Patients recently moved from another country (difficult to obtain history, different medications brands)				5	51.7	48.3				6	93.1	51.7
56	Patients lacking capacity to consent to medication administration	5	61.1	33.3	5	69.0	27.6	5	80.6	33.3	5	93.1	24.1
57	Patients aged >80 years				6	34.5	65.5				6	93.1	62.1
58	White blood cells levels outside reference range	6	44.4	55.6	6	44.8	55.2	5.5	83.3	50.0	5	93.1	41.4
59	Outstanding electrocardiogram	5	52.8	47.2	5	69.0	31.0	5.5	88.9	50.0	5	93.1	34.5
60	Warfarin	7	11.4	88.6	7	0.0	100.0	6	80.6	55.6	7	92.9	75.0
61	Intensive therapeutic drug monitoring drugs (e.g. phenytoin, carbamazepine)	6.5	20.6	79.4	6	7.1	92.9	6	73.5	64.7	6	92.9	78.6
62	Fall ≥ 1 in the preceding 3 months	6	48.6	51.4	6	39.3	60.7	5	85.7	40.0	5	92.9	28.6
63	More than 1 regular antidepressant prescribed				5	55.2	41.4				5	89.7	37.9
64	Prescribed a QTc prolonging medication (e.g chlorpromazine, quetiapine, amisulpride)	6	25.0	75.0	6	24.1	75.9	6	97.2	75.0	6	89.7	75.9
65	Patients with substance abuse	6	47.2	52.8	5	51.7	48.3	5	86.1	41.7	5	89.7	31.0
66	Patients with physical healthcare issues requiring follow‐up	6	44.4	55.6	6	34.5	65.5	5	91.7	41.7	5	89.7	41.4
67	Patients aged >65 years Round 2: Patients aged >70 years	5	58.3	41.7	5	75.9	24.1	5	75.0	41.7	5	89.7	20.7
68	Patients with undetermined allergy status	6	25.0	69.4	6	31.0	65.5	6	75.0	52.8	6	89.7	69.0
69	Patients planned for discharge/leave	5	52.8	36.1	5	75.9	24.1	6	86.1	63.9	6	89.7	58.6
70	T2/T3 renewal needed under the Mental Health Act Round 2: Patient prescribed medication prompting review of T2/T3 under the Mental Health Act	5	55.6	36.1	5	75.9	20.7	6	83.3	52.8	6	89.7	58.6
71	Patients who did not have VTE assessment Round 2: No VTE assessment for those prescribed antipsychotic	6	34.3	60.0	6	25.0	71.4	5	71.4	48.6	5	89.3	42.9
72	Patients in seclusion	6	31.4	68.6	6	32.1	67.9	6	85.7	54.3	6	89.3	60.7
73	Moderate hepatic impairment (liver function tests > ULN but <3× ULN)	6	32.4	64.7	6	25.9	70.4	6	70.6	58.8	6	88.9	59.3
74	Patients aged <18 years				6	44.4	55.6				6	88.9	51.9
75	Patients on the palliative care pathway	6	37.5	62.5	6	30.8	69.2	6	75.0	59.4	6	88.5	69.2
76	Patients aged <12 years				6	16.7	83.3				6	87.5	70.8
77	Alcohol detox medications (Pabrinex and/or Chlordiazepoxide)	6	27.8	66.7	6	24.1	72.4	6	83.3	63.9	6	86.2	69.0
78	More than 1 anxiolytic prescribed	5	50.0	47.2	5	58.6	41.4	5	83.3	44.4	5	86.2	27.6
79	Patients who self harm or have suicidal thoughts				6	41.4	58.6				6	86.2	51.7
80	Polypharmacy ≥5 regular medications	6	41.7	58.3	6	31.0	69.0	5	86.1	44.4	5	86.2	31.0
81	Anti‐cancer medications (e.g. azathioprine, fluorouracil)	6.5	9.4	90.6	6	3.7	96.3	5	68.8	43.8	6	85.7	53.6
82	Haemoglobin levels (CRP, HB1) outside reference range	5	65.6	34.4	5	85.2	14.8	5	71.0	22.6	5	85.2	14.8
83	More than 2 mood stabilisers prescribed	5	52.8	47.2	5	58.6	41.4	6	83.3	52.8	6	82.8	51.7
84	Patients who have recently stopped or started smoking				5	58.6	41.4				5	82.8	27.6
85	Patients prescribed off‐label medicines				5	62.1	37.9				5	79.3	24.1
86	Patients prescribed medicines by homecare	5	63.3	23.3	5	100.0	0.0	5	56.7	23.3	5	77.3	9.1
87	Phenytoin	6	27.8	69.4	6	31.0	69.0	5.5	66.7	50.0	5	75.9	48.3
88	CKD Stage > 2 (slightly reduced eGFR of 60 to 89 mL/min, with other signs of kidney damage)	5	52.8	41.7	5	62.1	34.5	5	69.4	36.1	5	75.9	24.1
89	Patients prescribed nonformulary medication	5	72.2	27.8	5	79.3	20.7	5	69.4	38.9	5	75.9	24.1
90	Antimuscarinics (e.g. procyclidine, oxybutynin)	5	66.7	27.8	5	82.8	13.8	5	69.4	44.4	5	72.4	17.2
91	Patients taking laxatives/have constipation				5	69.0	27.6				5	72.4	41.4
92	CKD Stage > 1 (normal eGFR above 90 mL/min, but other tests have detected signs of kidney damage)	5	57.1	28.6	5	72.4	17.2	5	60.0	31.4	5	72.4	10.3
93	Medicines compliance aid from community pharmacy e.g., dosette, pillpouch	5	77.8	16.7	5	96.6	3.4	5	61.1	27.8	5	72.4	3.4
94	Digoxin	6	37.1	62.9	6	25.0	75.0	5	54.3	25.7	5	71.4	14.3
95	Patients on self‐administration	5	66.7	22.2	5	86.2	10.3	5	61.1	30.6	5	69.0	10.3
96	Patients with new compliance aid requested	5	55.6	41.7	5	79.3	20.7	5	75.0	41.7	5	69.0	24.1
97	Antimicrobials or antivirals (e.g. vancomycin, itraconazole) Round 2: antimicrobials or antivirals (e.g. amoxicillin, nystatin)	6	34.3	65.7	6	42.9	57.1	5	57.1	34.3	5	67.9	32.1
98	Patients diagnosed with COVID	5	50.0	44.1	5	70.4	29.6	5	52.9	29.4	5	66.7	11.1
99	Paroxetine	5	75.0	19.4	5	86.2	10.3	5	52.8	19.4	5	65.5	6.9
100	Hospitalisation due to psychiatric condition ≥ 1 in the preceding year	5	61.1	33.3	5	75.9	24.1	5	66.7	33.3	5	65.5	24.1
101	Patient not spoken to by a pharmacy member within the past 7 days (acute) or fortnight (rehab)	5	66.7	25.0	5	72.4	20.7	5	58.3	27.8	5	65.5	17.2
102	Venlafaxine	5	77.8	16.7	5	86.2	13.8	5	55.6	16.7	5	62.1	10.3
103	Short course of steroids	5	55.6	41.7	5	62.1	34.5	5	55.6	16.7	5	62.1	10.3
104	Contraceptives (e.g. Yasmin, Evra)	4	74.3	8.6	4	89.3	3.6	5	51.4	20.0	5	57.1	10.7
105	Amiodarone	6	47.1	52.9	6	32.1	67.9	4	47.1	23.5	5	53.6	10.7
106	Theophylline	6	47.1	52.9	5	51.9	48.1	4	44.1	20.6	4	48.1	7.4
107	Hydrocortisone tablets (for adrenal insufficiency/Addison's disease)	6	42.9	57.1	6	32.1	67.9	4	45.7	34.3	4	46.4	25.0
108	Patient taking antidementia medications	4	72.2	16.7	4	82.8	10.3	5	55.6	27.8	4	44.8	6.9
109	Aminophylline	5.5	46.9	50.0	5	53.8	46.2	4	45.5	27.3	4	34.6	11.5
110	Patient taking antidepressants	4	69.4	19.4	4	82.8	6.9	5	52.8	33.3	4	34.5	6.9
111	Patient with daily aseptic needs e.g. on total parenteral nutrition, antibiotic infusion	6	19.2	80.8	6	19.0	81.0	4	44.0	32.0	4	33.3	19.0
112	Hospitalisation due to nonpsychiatric condition >1 in the preceding year	5	62.9	31.4	5	82.1	17.9	4	40.0	14.3	4	32.1	7.1
113	Digoxin, amiodarone loading	6	20.7	79.3	6	8.0	92.0	3.5	39.3	32.1	4	32.0	16.0
114	Desmopressin (for cranial diabetes insipidus)	5	53.3	43.3	5	66.7	29.2	4	45.2	29.0	4	32.0	20.0
115	Diuretics	5	77.8	16.7	5	86.2	10.3	4	33.3	8.3	4	31.0	10.3
116	Type I, III or IV antiarrhythmics	5	53.3	43.3	5	75.0	25.0	4	44.8	24.1	4	25.0	8.3
117	Magnesium supplements (e.g. magnesium glycinate, magnesium aspartate)	4	75.8	9.1	4	85.7	7.1	4	27.3	6.1	4	17.9	3.6
118	Angiotensin converting enzyme inhibitors or angiotensin receptor inhibitors	4	80.6	5.6	4	86.2	6.9	4	30.6	5.6	4	17.2	0.0
119	Tretinoin	5	51.7	41.4	5	62.5	37.5	3.5	33.3	10.0	4	16.7	8.3
120	Diltiazem	5	70.6	20.6	5	81.5	11.1	3	26.5	5.9	3	7.4	0.0
121	Selective oestrogen receptor modulators (e.g. tamoxifen, raloxifene)	4	63.6	30.3	4	92.6	0.0	3	21.2	12.1	3	3.7	0.0
122	Hormone replacement therapy (e.g. Premique, Premarin)	3.5	63.9	8.3	3	79.3	3.4	3	25.0	5.6	3	3.4	0.0
123	Bisphosphonates (e.g. alendronate, risedronate)	4	68.6	11.4	4	92.9	0.0	3	17.1	5.7	3	0.0	0.0

Abbreviations: BNF, British National Formulary; CKD, chronic kidney disease; eGFR, estimated glomerular filtration rate; JEJ, jejunostomy tube; NG, nasogastric tube; PEG: percutaneous endoscopic gastrostomy; ULN, upper limit of normal; VTE, venous thromboembolism.

^**#**^ Low‐risk if ≥75% rated 1–2, medium‐risk if ≥75% rated 3–5 and high‐risk if ≥75% rated 6–7.

^##^ Included in the tool if ≥75% rated importance 6–7 or ≥85% rated importance 5–7.

^a^ An adverse drug reaction occurred that may have contributed to or resulted in patient harm.

^b^ An interaction occurred that either requires action to be taken to avoid harm or may have contributed to or resulted in patient harm.

^c^ Referring to related medication management issues.

The traffic light classification system (red, amber and green) was the most preferred by 58.6% (*n =* 17/29) of the participants and the second most preferred by 37.9% (*n =* 11/29) in the round 2 ranking exercise (Table 3). Hence, this classification system was used along with the 82 risk indicators achieving consensus to develop a draft for the IMPACT tool. Ten similar risk indicators were merged together to reduce the complexity of the tool (e.g. chronic kidney disease [CKD] stages were combined into 2 groups: CKD stage >3b and CKD stage ≤ 3b) and 1 risk indicator was excluded by stakeholders. Finally, 71 risk indicators were used to develop the tool. A flowchart illustrating the refinement process for the risk indicators can be viewed in Appendix 6.

**TABLE 3:** Results of classification systems ranking in Delphi study 1.

Classification system	Ranking		
	1	2	3
**Traffic light (red, amber and green)**	17	11	1
**Levels (1–4)**	11	11	8
**Only high‐risk indicators**	1	7	20

Ranking was from the most preferred (1) to the least preferred (3).

### 4.2 Delphi study 2

Forty‐three experts were invited to take part in Delphi study 2 questionnaire of which 32 (74.4%) completed the first round and 30 (93.8%) completed the second round. Similarly to Delphi study 1, participants had between 3 to over 20 years of experience working in mental health but this questionnaire was only distributed to pharmacists and pharmacy technicians (see Table 1). The majority had their recent experience at an NHS organization based in England (81.3%, *n =* 26/32), followed by Scotland (15.6%, 5/32), with 1 in Wales and none was working in an organization from Northern Ireland.

A total of 29 statements about the use of the tool were included in round 1 along with open‐ended questions exploring participants' views on the draft tool. From these, there was agreement on 12 (41.3%) statements in round 1 and 13 (44.8%) statements in round 2 (Table 4). None of the 9 statements focusing on the frequency of review for each risk category reached agreement in round 1, with only 1 out of 10 reaching agreement in round 2. The ranking questions for frequencies of pharmacy team review included in round 2 helped the research team make a decision on which frequency to use for each risk group. The most preferred frequency of review was every 1–2 days for the high‐risk group, every 2–4 days for the medium‐risk group and once every working week for the low‐risk group (Table 5).

**TABLE 4:** Results of both rounds of Delphi study 2.

	Statements	Round 1						Round 2					
		*n*	Median	Mean	1–4%	5–7%	6–7%	*n*	Median	Mean	1–4%	5–7%	6–7%
1	Training should include some worked examples for the model.	32	7	6.6	0.0	100.0	96.9	30	7	6.6	0.0	100.0	100.0
2	Training in how to use the prioritization tool should be available for staff new to mental health services.	32	7	6.5	3.1	96.9	90.6	30	7	6.6	0.0	100.0	96.7
3	Training should include the benefits of using the model for staff whether they are new or experienced in mental health.	32	6	6.2	3.1	96.9	81.3	30	6	6.3	0.0	100.0	93.3
4	Clinical judgement should be incorporated in the prioritization model using a specific *other* option that pharmacy team members can select within each risk category, with a compulsory text comment field to provide any further details.	32	6	6.1	6.3	93.8	78.1	30	6	6.2	0.0	100.0	90.0
5	The model should be introduced to staff by explaining the benefits of using the tool, previous experiences and how the model was developed.	32	6	6.3	0.0	100.0	81.3	30	6	6.4	0.0	100.0	90.0
6	The patient prioritization model should be used by both pharmacists and pharmacy technicians alike.	32	7	6.4	3.1	96.9	90.6	30	7	6.4	0.0	100.0	86.7
7	At least 1 training session should be delivered to pharmacy team members prior implementing the patient prioritization model.	32	7	6.6	3.1	96.9	96.9	30	7	6.6	3.3	96.7	96.7
8	Training should include the importance of balancing standardisation and clinical judgement when prioritizing patients.	32	6.5	6.3	3.1	96.9	87.5	30	6	6.3	3.3	96.7	93.3
9	Training should include some feedback from previous users of the tool.	32	6	6.0	6.3	93.8	81.3	30	6	6.2	3.3	96.7	93.3
10	Individual patients identified who have >5 amber criteria should be moved to the red category.	32	6	5.8	18.8	81.3	75.0	30	6	5.9	3.3	96.7	76.7
11	Individual patients identified who have >4 amber criteria should be moved to the red category.	32	6	5.6	12.5	87.5	62.5	30	6	5.7	3.3	96.7	70.0
12	The patient prioritization model can be used during medication reconciliation on admission.	32	6	6.0	6.3	93.8	68.8	30	6	5.8	6.7	93.3	63.3
13	Individual patients identified as low risk following completion of the prioritization model should be reviewed once every working week or more frequently based on referral.	32	6	5.1	25.0	75.0	53.1	30	6	5.6	10.0	90.0	76.7
14	^a^ Individual patients identified as high risk following completion of the prioritization model should be reviewed by or discussed with an experienced pharmacist in the relevant area.	32	5	5.2	25.0	75.0	46.9	30	5	5.2	16.7	83.3	46.7
15	Individual patients identified who have >3 amber criteria should be moved to the red category.	32	5	5.1	28.1	71.9	31.3	30	5	5.0	16.7	83.3	20.0
16	Annual training for all pharmacy teams is needed to review and update the use of the prioritization model.	32	6	5.3	25.0	75.0	53.1	30	6	5.4	20.0	80.0	56.7
17	Individual patients identified as high risk following completion of the prioritization model should be reviewed every 1–2 days.	32	5	5.0	28.1	71.9	40.6	30	5	5.0	23.3	76.7	30.0
18	Individual patients identified as medium risk following completion of the prioritization model should be reviewed twice a week.	32	5	4.5	43.8	56.3	31.3	30	5	4.8	26.7	73.3	23.3
19	Different prioritization criteria should be used by pharmacy technicians to refer patients for pharmacist review.	32	5	4.6	43.8	56.3	37.5	30	5	4.5	33.3	66.7	30.0
20	For new admissions, staff members completing the patient prioritization model may skip the remaining criteria once they marked at least 1 red criterion. Marking 1 red criterion will identify the patient as a high‐risk patient and skipping the rest of criteria will speed up the prioritization process.	32	5	4.6	43.8	56.3	37.5	30	5	4.7	33.3	66.7	26.7
21	^a^ Individual patients identified as medium risk following completion of the prioritization model should be reviewed by or discussed with an experienced pharmacist in the relevant area.	32	5	4.8	40.6	59.4	40.6	30	5	4.6	36.7	63.3	26.7
22	Individual patients identified as high risk following completion of the prioritization tool should be reviewed daily.	32	5	4.7	40.6	59.4	34.4	30	5	4.6	36.7	63.3	26.7
23	The patient prioritization tool can be used before medication reconciliation on admission.	32	5	4.7	37.5	62.5	34.4	30	5	4.4	40.0	60.0	23.3
24	Individual patients identified as medium risk following completion of the prioritization tool should be reviewed every 2–4 days.	32	5	4.3	46.9	53.1	25.0	30	5	4.5	40.0	60.0	10.0
25	^b^ Individual patients identified as high risk following completion of the prioritization tool should be reviewed 3 times a week.							30	5	4.3	43.3	56.7	20.0
26	Individual patients identified as low risk following completion of the prioritization tool should be reviewed every 3–7 days or upon referral.	32	5	4.3	43.8	56.3	21.9	30	5	4.4	43.3	56.7	13.3
27	Different prioritization criteria should be used by pharmacy technicians to categorize patients for pharmacy technician review.	32	5	4.2	46.9	53.1	31.3	30	4	3.9	56.7	43.3	13.3
28	Individual patients identified as medium risk following completion of the prioritization tool should be reviewed every 2–3 days.	32	4	4.2	53.1	46.9	18.8	30	4	4.1	60.0	40.0	6.7
29	Individual patients identified as medium risk following completion of the prioritization tool should be reviewed every 1–2 days.	32	4	3.9	65.6	34.4	12.5	30	4	3.9	63.3	36.7	10.0
30	Individual patients identified as low risk following completion of the prioritization tool should be reviewed every 14 days or upon referral.	32	3	3.3	75.0	25.0	9.4	30	3	3.2	83.3	16.7	10.0

*Note*: A statement was included in the tool if ≥75% rated it 6–7 or ≥85% rated it 5–7.

^a^ These statements were modified in round 2, the original statements used in round 1 were: *“Individual patients identified as high‐risk/medium‐risk following completion of the prioritization tool should be reviewed by or discussed with a senior pharmacist”*.

^b^ Statement was added in round 2 (was not used in round 1).

**TABLE 5:** Results of ranking question for the frequency of review in Delphi study 2.

Risk category	Frequency of review	Ranking			
		1	2	3	4
**High risk**	Daily	6	3	20	
	**Every 1–2 days**	12	17	0	
	3 times a week	11	9	9	
**Medium risk**	Every 1–2 days	3	3	4	19
	Every 2–3 days	4	11	12	2
	**Every 2–4 days**	11	8	9	1
	Twice a week	11	7	4	7
**Low risk**	**Once every working week or more frequently based on referral**	19	10	0	
	Every 3–7 days or upon referral	9	18	2	
	Every 14 days or upon referral	1	1	27	

*Note*: Ranking was from the most preferred (1) to the least preferred (3 or 4).

For the second part of the questionnaire, participants provided comments and suggestions on the draft tool, which was presented to them through screenshots and a PDF file. After the second round, there was an agreement on the final tool with minor suggestions (a summary of suggestions is presented in Appendix 7 and the final tool is presented in Appendix 8). Other than that, panel members were generally happy with the tool.

*“I think this will be really useful and is going to be a helpful method of prioritising patients that require more attention. It seems as if it will be quite quick to use, particularly when people have become familiar with it, which will help with embedding this into routine practice”* (P27, pharmacist).

Participants also expressed positive views towards the design of the tool shown to them as a draft mock up.

*“Good that it's colour coded and tick box for minimal time spent completing and also reading”* (P8, pharmacist).

## 5 DISCUSSION

This study presents the first evidence‐ and consensus‐based patient prioritization tool designed specifically for UK inpatient pharmacy teams. The IMPACT tool was rigorously developed through the integration of the available evidence, knowledge of current prioritization approaches within UK mental health units and expert opinion. Panel members showed enthusiasm toward using this tool in the future, further emphasizing its potential value in practice. The tool has been designed with the potential to contribute to increased patient safety and optimal pharmacy service delivery in future.

In Delphi study 1, experts agreed on including 66.7% (*n =* 82/123) of the risk indicators in the tool. Despite using a strict definition of consensus, a high percentage of risk indicators was agreed upon which may be expected considering the risk averse nature of pharmacists reported in other studies.^32^, ^33^ An even higher percentage (84.4%) was reported in a similar Delphi study focused on risk indicators for medication‐related problems in acute care.^24^ By contrast, a lower percentage (44.8%, *n =* 13/29) of the statements reached agreement in Delphi study 2. This might be partially attributed to the 9 statements that focused on the frequency of review, where pharmacy team members had different opinions based on their local sites' capacity and working practices. This highlights the importance of adapting the prioritization tool as needed by different sites.

Many risk indicators that were agreed on differed from those included in an acute care pharmaceutical patient prioritization tool.^24^ For example, there was no agreement on including phenytoin in our study but agreement was achieved in acute care.^24^ The high‐risk medicines list in acute care included a greater range of medications such as analgesics, chemotherapy, immunosuppressants and some intravenous medicines.^24^ Additionally, some mental health specific risk indicators agreed on by experts in our study were not included in the acute care tool^24^ such as the use of zuclopenthixol acetate and patients in seclusion. Moreover, review of patients in acute care was more frequent (high risk = daily, moderate risk = every 1 or 2 days and low risk: twice weekly) and included a narrower window for review on admission (6–12 h and up to 24 h for high‐risk and 24 h for moderate‐risk patients).^24^ These differences between mental health and acute care risk indicators emphasise the importance of having a prioritization tool that is specific to the context of mental health care.

Many participants commented on risk indicators and statements, adding valuable opinions and insights which were further considered in the second round of each Delphi study. Comments showed the complexity of risk indicators and the difficulty in rating them, considering their broad dimensions and future application. They also emphasised the importance of using clinical judgement alongside the tool criteria. Future research could focus on evaluating the identified risk indicators by measuring their accuracy in identifying high‐risk patients. For example, a cohort study was previously used to measure the relationship between potential risk indicators for drug‐related problems and the occurrence of moderate or severe drug‐related problems in general hospitals which identified 11 risk indicators predicting the occurrence of drug‐related problems.^34^

Participants liked the simplicity of the design and the use of colour coding and tick boxes as it was thought to facilitate engagement with and use of the tool. Similar findings were reported in a systematic review focusing on the implementation of pharmacy services, which found that the increased complexity of an intervention is linked to a need for more resources, thereby hindering the implementation of the intervention.^35^ Additionally, referencing the evidence behind the tool development was advocated by 1 participant, which might influence changing staff behaviour—a main factor for successful implementation of complex interventions.^36^ The use of an electronic format was preferred by all participants for ease of use and sharing, similar to many other organizations that have chosen to develop electronic prioritization tools.^19^, ^21^ However, the use of electronic tools may introduce confidentiality issues and technical difficulties, and may limit its use to certain electronic prescribing systems. While we may overcome the first 2 issues with technological advancement, the main challenge remains with adapting the tool to diverse electronic systems across organizations. The challenge relates to both the number of different systems that are currently used (20 suppliers were reported by NHS digital in 2023^37^) and the differences across the systems.

A Delphi study was used to develop the tool which has strengths such as using controlled feedback which adds an element of objectivity,^38^ and avoiding the *halo effect*—where participants' opinion is affected by a certain individual—through the anonymity of participants.^39^ Additionally, a small group of key stakeholders were involved throughout the development process including 2 patient representatives with lived experience in mental health wards. The expert panel in Delphi study 1 included psychiatrists and academics along with pharmacists to ensure a wide perspective was captured. By contrast, only pharmacists and pharmacy technicians were included in Delphi study 2 as it focused on practical aspects that relate to tool users. Moreover, the Delphi questionnaires were developed using an international systematic review^22^ and national expertise.^21^ However, the study has some limitations such as the lack of expert representation from Northern Ireland. Additionally, while adding an *outside my expertise* option in Delphi study 1 limited guessing and captured sincere uncertainty, it posed another challenge as sometimes experts avoided rating uncommon risk indicators such as *total parenteral nutrition*. However, despite some experts choosing *outside my expertise* for indicators such as this, there was clear agreement among experts who rated them.

In conclusion, this study has developed a novel, evidence‐ and consensus‐based patient prioritization tool for use by mental health inpatient pharmacy teams in the UK. Implementation of the IMPACT tool within mental health hospital pharmacy services might improve service efficiency and safety of patient care. Acceptability testing of the tool by mental health pharmacy teams could be initially conducted to gather insight on the tool's usability, relevance and potential impact. This could be followed by a before and after feasibility testing study to explore the potential impact of the tool on identifying high‐risk patients and improving patient outcomes. Once feasibility is proved, a randomised controlled trial may be conducted to explore the efficacy of the tool.

## AUTHOR CONTRIBUTIONS

This study was part of FQA's PhD project supervised by PJL and RNK. All the authors contributed to the conceptualisation of the research idea and development of the protocol. Data collection, analysis, and writing of the manuscript were led and performed by FQA, supported by PJL, RNK, and PB. All authors read and approved the final manuscript.

## CONFLICT OF INTEREST STATEMENT

The authors declare that they have no competing interests.

## ETHICS APPROVAL

Ethical approval was obtained from the University of Manchester ethics committee (No. 16447).

## Supporting information


**TABLE S1** Results of round 1 of Delphi study 1.
**TABLE S2** Results of round 2 of Delphi study 1.

## Data Availability

The datasets used and/or analysed during the current study are available from the corresponding author on reasonable request.
