# Supplementary material for: Developing the Inpatient Mental Health Pharmaceutical Assessment and Care Tool (IMPACT) for use by UK mental health pharmacy teams—a modified Delphi study
Source: Br J Clin Pharmacol. 2025 May 23;91(10):2836–53. doi: 10.1002/bcp.70083 (PMC12464646; doi:10.1002/bcp.70083)
Supplement: Supplementary file 1 — TABLE S1 Results of round 1 of Delphi study 1. TABLE S2 Results of round 2 of Delphi study 1. [file BCP-91-2836-s001.docx]

**Appendices**

**Study title:** Developing the Inpatient Mental Health Pharmaceutical Assessment and Care Tool (IMPACT) for use by UK mental health pharmacy teams – a modified Delphi study

**Journal:** British Journal of Clinical Pharmacology

**Authors:** Fatima Q. Alshaikhmubarak^1^, Richard N. Keers^1,2,3^, Petra Brown^1,3^, Penny J. Lewis^1,2,4^

**Author Affiliations**

1. Division of Pharmacy and Optometry, The University of Manchester, Manchester, UK

2. NIHR Greater Manchester Patient Safety Research Collaboration, Manchester, UK

3. Optimising Outcomes with Medicines (OptiMed) Research Unit, Pennine Care NHS Foundation Trust.

4. Manchester University NHS Foundation Trust, Manchester, UK

Corresponding author:

Fatima Alshaikhmubarak, [fatema.alshaikhmubarak@manchester.ac.uk](mailto:fatema.alshaikhmubarak@manchester.ac.uk)

**Appendix.1**

| **Guiding Principles** |
| --- |
| 1. Ensure the patients’ voice is included. |
| 1. Ensure clarity of risk indicators to allow pharmacy members to identify them easily, e.g. define what is meant by a ‘significant drug interaction’. |
| 1. Combine similar indicators, e.g. combine ‘physical healthcare issues requiring follow-up’ and ‘patients with significant physical health needs’. |
| 1. Ensure relevance to the aim of the tool, e.g. ‘critical incidents’; these have already occurred, the questionnaire aims to identify risk indicators not previously arising drug related problems. |
| 1. Group medications together, e.g. hormone replacement therapy medications. |
| 1. Remove indicators if they are captured elsewhere, e.g. ‘change in concomitant medications’ is captured under ‘unverified medications’. |
| 1. Ensure relevance to mental health, e.g. remove ‘intravenous medications’ as these are seldom used in mental health settings. |
| 1. Ensure practicality, e.g ‘cognitive impairment’ is difficult to measure. |
| 1. Ensure applicability, e.g. ‘prescription type’ and ‘prescriber grade’ include nearly all types and grades, hence all patients will be prioritised. |

**Appendix.2**

After meeting with stakeholders, one risk indicator was added based on their suggestion:

“Patient not spoken to by a pharmacy member within the last rolling working week (acute) or fortnight (rehab)”

On the other hand, several risk indicators were excluded or modified. Excluded and modified risk indicator could be seen in the table below along with their origin and reason for exclusion/modification:

| **High risk drugs** | | |
| --- | --- | --- |
| **Drug name** | **Origin** | **Justification** |
| Insulin IF: Not suitable to self administer, Insulin switched as per policy, Blood glucose < 4mmol/l (Hypo)) | Existing tools | Collated with insulin. |
| Palliative care medicines | Existing tools | Patient in palliative care pathway captured in patient related factors |
| BUSULFAN | Existing tools | These antineoplastics are commonly given intravenously. It was agreed in the stakeholders meeting that intravenous medications are seldom used in mental health units. |
| CYCLOPHOSPHAMIDE | Existing tools |  |
| ETOPOSIDE | Existing tools |  |
| FLUDARABINE | Existing tools |  |
| HYDROXYCARBAMID | Existing tools |  |
| IDARUBICIN | Existing tools |  |
| MELPHALAN | Existing tools |  |
| METHOTREXATE | Existing tools |  |
| LOMUSTINE | Existing tools |  |
| MITANE - Mitotane | Existing tools |  |
| PROCARBAZINE | Existing tools |  |
| TEMOZOLOMIDE | Existing tools |  |
| PAMIDRONATE | Existing tools | These bisphosphonates are given intravenously as above. |
| ZOLEDRONIC acid | Existing tools |  |
| Daptomycin | Existing tools | These antimicrobials are intravenous. It was agreed in the stakeholders meeting that intravenous medications are seldom used in mental health units. |
| Meropenem | Existing tools |  |
| Teicoplanin | Existing tools |  |
| Tigecycline | Existing tools |  |
| Doripenem | Existing tools |  |
| Amphotericin B liposomal | Existing tools |  |
| Micafungin | Existing tools |  |
| Ertapenem | Existing tools |  |
| Imipenem | Existing tools |  |
| Ticarcillin and clavulanate potassium | Existing tools |  |
| Aztreonam | Existing tools |  |
| Temocillin | Existing tools |  |
| Quinupristin/dalfopristin | Existing tools |  |
| Amphotericin B lipid complex | Existing tools |  |
| Piperacillin | Existing tools |  |
| Anidulafungin | Existing tools |  |
| Amikacin | Existing tools |  |
| Caspofungin | Existing tools |  |
| Colistimethate Sodium | Existing tools |  |
| Dalbavancin | Existing tools |  |
| Pentamidine | Existing tools |  |
| Streptomycin | Existing tools |  |
| Telavancin | Existing tools |  |
| Tobramycin | Existing tools |  |
| PARACETAMOL 1000 mg in 100ml Intravenous Infusion | Existing tools | Intravenous as above. |
| PARACETAMOL - IN RECOVERY ONLY 1000 mg in 100mL IV Infusion | Existing tools |  |
| ADULO | Existing tools | There is no medication found with this name |
| New medication for substance misuse | Existing tools | Captured with unverified medications |
| **Drug related risk indicators** | | |
| **Adverse drug reactions/ Side effects** | **Origin** | **Justification** |
| Unresolved side-effects that require management | Existing tools | Collated into one. |
| Significant adverse drug reaction (ADR) if noted ADR e.g. recent fall or prolonged QTc >500ms. | Existing tools |  |
| Significant adverse drug reaction (ADR) if NO current indication of ADR e.g. history of falls or prolonged QTc- monitor for any changes to medication. | Existing tools |  |
| **Drugs interaction** |  |  |
| Lithium with interacting medications that have already been screened | Existing tools | Captured in high risk medicines. |
| Interactions | Existing tools | Collated into one. |
| Significant drug interaction if indication of toxic/ subtherapuetic effect resulting from interaction. | Existing tools |  |
| Significant drug interaction if NO indication of toxic/ subtherapuetic effect resulting from interaction. | Existing tools |  |
| More than one regular antipsychotic prescribed | Existing tools | Each statement regarding certain medication class were collated together. Agreement as part of treatment plan was removed due to impracticality. |
| More than one regular antipsychotic (including depot) which has not been agreed as part of a treatment | Existing tools |  |
| More than one regular antipsychotic (including depot)  which has been agreed as part of a treatment plan | Existing tools |  |
| More than one antidepressant *which has not been agreed as part of a treatment* | Existing tools |  |
| More than one antidepressant *which has been agreed as part of a treatment plan* | Existing tools |  |
| More than one hypnotic *which has not been agreed as part of a treatment* | Existing tools |  |
| More than one hypnotic *which has been agreed as part of a treatment plan* | Existing tools |  |
| More than one anxiolytic *which has not been agreed as part of a treatment* | Existing tools |  |
| More than one anxiolytic *which has been agreed as part of a treatment plan* | Existing tools |  |
| More than two mood stabilisers *which has not been agreed as part of a treatment* | Existing tools |  |
| More than two mood stabilisers *which has been agreed as part of a treatment plan* | Existing tools |  |
| more than two mood stabilisers | Existing tools |  |
| **Dose related issues** |  |  |
| Unusual doses | Existing tools | Captured under unverified medications |
| cross tapering psychotropics | Existing tools | Captured under > 1 antipsychotic above. |
| High Dose Antipsychotic Therapy (above 100% BNF maximum) which has not been agreed as part of a treatment plan | Existing tools | These were collated. |
| High Dose Antipsychotic Therapy (above 100% BNF maximum) which has been agreed as part of a treatment plan | Existing tools |  |
| Increase of a regular psychotropic within 7 days of the last increase *(unless as part of a dose titration regimen) which has not been agreed as part of a treatment* | Existing tools |  |
| Increase of a regular psychotropic within 7 days of the last increase *(unless as part of a dose titration regimen) which has been agreed as part of a treatment plan* | Existing tools |  |
| Any single drug above BNF limits *(unless planned detoxification) which has not been agreed as part of a treatment* | Existing tools |  |
| Any single drug above BNF limits *(unless planned detoxification) which has been agreed as part of a treatment plan* | Existing tools |  |
| HDAT (high dose antipsychotic therapy) | Existing tools |  |
| Patients requiring high dose antipsychotic monitoring (HDAT) that is overdue. | Existing tools |  |
| **High risk medicines/ dose change** |  |  |
| Routine TDM | Existing tools | Collated into TDM levels, and high risk medicines captured in the list. |
| Therapeutic drug monitoring where requested for specific reason | Existing tools |  |
| High risk medicine / medicine requiring TDM if some indication of toxic or subtheraputic effect (e.g. SACTs, cytotoxics, digoxin, lithium, phenytoin, theophylline, vancomycin, warfarin, valproate in women of childbearing potential etc) | Existing tools |  |
| High risk medicine / medicine requiring TDM if NO indication of toxic or subtheraputic effect (e.g. SACTs, cytotoxics, digoxin, lithium, phenytoin, theophylline, vancomycin, warfarin, valproate in women of childbearing potential etc) | Existing tools |  |
| Critical Medicines - Where clinically unscreened or requires supply | Existing tools |  |
| Patients receiving ‘high risk’ or and/or ’time critical’ medications | Existing tools |  |
| Starting high risk medicines | Existing tools | Captured under unverified and high risk medicines. |
| Valproate Alert | Existing tools |  |
| Clozapine titration | Existing tools |  |
| Clozapine initiation requests | Existing tools |  |
| Anything around clozapine (levels, orders, or pharmacy interventions) | Existing tools |  |
| Clozapine dose change | Existing tools |  |
| Lithium dose change | Existing tools |  |
| Prescribed a QTc prolonging medication | Existing tools | Collated into one and examples added. |
| Prescribed a combination of QTc prolonging medication of which at least one is newly started or dose increase | Existing tools |  |
| Short term use of antipsychotics/ benzodiazepines in delirium/ agitation for patients with contra-indication/ cautions for use of antipsychotics e.g. Parkinsons, Lewy body dementia etc. | Existing tools | Captured under unverified and high risk medicines. |
| Short term use of antipsychotics/ benzodiazepines in delirium/ agitation for patients with NO obvious contra-indication to pharmacological management. | Existing tools |  |
| Any prescription for Zuclopentixol Acuphase *which has not been agreed as part of a treatment* | Existing tools | Captured earlier under unverified and high risk medicines and QTc. |
| Any prescription for Zuclopentixol Acuphase *which has been agreed as part of a treatment* | Existing tools |  |
| Any IM dose in 24 hours of lorazepam, olanzapine, haloperidol, promethazine or aripiprazole *which has not been agreed as part of a treatment* | Existing tools |  |
| Any IM dose in 24 hours of lorazepam, olanzapine, haloperidol, promethazine or aripiprazole *which has been agreed as part of a treatment plan* | Existing tools |  |
| Regular prescription of any hypnotic for more than 4 weeks *which has not been agreed as part of a treatment* | Existing tools |  |
| Regular prescription of any hypnotic for more than 4 weeks *which has been agreed as part of a treatment plan* | Existing tools |  |
| Regular prescription of any anxiolytic for more than 4 weeks *which has not been agreed as part of a treatment* | Existing tools |  |
| Regular prescription of any anxiolytic for more than 4 weeks *which has been agreed as part of a treatment plan* | Existing tools |  |
| On more than one drug that could prolong the QTc interval *which has not been agreed as part of a treatment* | Existing tools |  |
| On more than one drug that could prolong the QTc interval *which has been agreed as part of a treatment plan* | Existing tools |  |
| Restricted medicines: unverified or Verification under review prescriptions | Existing tools | Captured earlier under unverified and high risk medicines. |
| High alert medicines: unverified or Verification under review prescriptions | Existing tools |  |
| Methadone prescriptions: unverified or Verification under review prescriptions. | Existing tools |  |
| Lithium prescriptions: unverified or Verification under review prescriptions. | Existing tools |  |
| Immunosuppressant medicines: unverified or Verification under review prescriptions. | Existing tools |  |
| Opiate prescriptions: unverified or Verification under review prescriptions. | Existing tools |  |
| Anticoagulant prescriptions: unverified or Verification under review prescriptions. | Existing tools |  |
| Anticoagulant prescriptions: verified. | Existing tools |  |
| Gentamicin/Vancomycin prescriptions  (Intra route) | Existing tools |  |
| Oral Potassium or Phosphate prescriptions >5 days | Existing tools | These were from a prioritisation tool developed for acute care, they are not relevant in a mental health setting. Some are relevant but these are captured under high risk medicines. |
| Calcium Resonium prescriptions >3 days | Existing tools |  |
| IV Paracetamol prescriptions >3 days | Existing tools |  |
| Pabrinex prescriptions >5 days | Existing tools |  |
| Diazepam prescriptions >5 days | Existing tools |  |
| Prednisolone (high dose > 30 mg) prescriptions >5 days | Existing tools |  |
| Metoclopramide (regular not PRN) prescriptions >5 days | Existing tools |  |
| Oral antibacterial prescriptions >5 days | Existing tools |  |
| Injected antibacterial prescriptions >2 days | Existing tools |  |
| Eye/ear/mucosal/buccal antibacterial prescriptions >7 days | Existing tools |  |
| Topical antibacterial preparations >10 days | Existing tools |  |
| Non-psychotropic medications | Literature | Not relevant to the aim of the tool we are trying to develop. |
| Non-oral route medications | Literature |  |
| Controlled drugs on discharge | Existing tools | Captured under high risk medicines. |
| **Patient related risk indicators** | | |
| **Kidney function** | **Origin** | **Justification** |
| Acute renal impairment (e.g.AKI>1) | Existing tools | These were collated and reorganised with the help of NHS stages guide. |
| Acute kidney injury (urea ≥ 10, creatine ≥ 30 from baseline) on potentially nephrotoxic medicines | Existing tools |  |
| Acute kidney injury (urea ≥ 10, creatine ≥ 30 from baseline) NOT on potentially nephrotoxic medicines | Existing tools |  |
| Chronic renal impairment (CKD) | Existing tools |  |
| Severe chronic renal impairment (Stage 4 or 5 CKD) | Existing tools |  |
| Severe chronic renal impairment (Est. CrCl ≤ 30ml/min) on medications requiring close adjustment | Existing tools |  |
| Severe chronic renal impairment (Est. CrCl ≤ 30ml/min) NOT on medications requiring close adjustment | Existing tools |  |
| CrCl (manual input) | Existing tools |  |
| GFR (manual input) | Existing tools |  |
| **Medical conditions** |  |  |
| Behavioural and Psychological Management of Dementia and prescribed psychotropics to manage | Existing tools | This was discussed with stakeholders and it was agreed that this is only applicable when not on a dementia ward, so that was kept and this one excluded. |
| Cognitive impairment | Literature | Not practical, difficult to measure. |
| Decreased personal care activities | Literature |  |
| Increased clinical global impression (CGI) at admission | Literature |  |
| Increased charlson comorbidity index (CCI) | Literature |  |
| Psychiatric disorder | Literature | Not applicable as many people in mental health wards will have these diseases. |
| F1, F2, and F3 diagnosis | Literature |  |
| Schizophrenia | Literature |  |
| Somatic diagnosis | Literature |  |
| Diagnosis of organic brain disease | Literature |  |
| Diabetes | Existing tools | This is captured in high risk meds: Insulin |
| Frailty (consider sedative medicines in over 65 years) | Existing tools | This is captured in age>65. |
| Physical healthcare issues requiring follow-up/intervention/ ongoing monitoring. | Existing tools | These were collated. |
| Patients with significant physical health needs/long term conditions requiring daily follow up· | Existing tools |  |
| Patients with stable long term conditions i.e. diabetes | Existing tools |  |
| Thromb | Existing tools | Captured in VTE. |
| Patients over the age of 65 or at risk of cognitive impairment with an anticholinergic burden (ACB) score >3. | Existing tools | Captured in age >65. |
| **Admission/ Transfer** |  |  |
| Transfer of care | Existing tools | These are all captured in unverified medications and medicinces reconcilliation. Additionally, transfer of care or admission would be a time when the tool will be used anyways and patient will be prioritised then based on the other factors. |
| Transferred to PICU (Psychiatric intensive care unit) | Existing tools |  |
| Patients who have returned from acute hospital following period of leave for physical health intervention. | Existing tools |  |
| New patients | Existing tools |  |
| Newly admitted prior to initial senior medical review on the ward | Existing tools |  |
| New patients prescribed non-standard regimens of psychotropic medicine | Existing tools |  |
| new patients prescribed medicines for physical health problems | Existing tools |  |
| **pharmacy issues** |  |  |
| MAR chart – both new and existing | Existing tools | These are part of existing processes such as medicines reconcilliation and discharge planning . |
| Instalment dispensing e.g. methadone, diazepam, antidepressants – please record details | Existing tools |  |
| Counselling - GASS/GASS-C, LISERs, discharge, lithium | Existing tools |  |
| All patients/carers referred by the ward team or self-referred for medication counselling. | Existing tools |  |
| **MHA** |  |  |
| Patients under section 3 or 37 due for SOAD review. | Existing tools | These were collated, SOAD is part of T3 form and it was separated in 2 indicators, new or renewal T2/T3. |
| Mental Health Act: I/E/S/C (Informal, emergency detention, short-term, or compulsory detention) | Existing tools |  |
| Mental Health Act Status (e.g.consent to treat paperwork) | Existing tools |  |
| T2/T3 due: | Existing tools |  |
| **Covert/ self administration** |  |  |
| New covert administration (at least week 1 and 2) | Existing tools | Collated into one. |
| Established covert administration. | Existing tools |  |
| **Demographics** |  |  |
| Weight | Existing tools | Not risk indicators, calculation might be needed for other reason such as CrCl. |
| Height | Existing tools |  |
| Increased age # | Literature | Collated with age > 65. |
| Female | Literature | Was only found significant in one study in the literature compared with many studies not finding significance. Additionally, it is not relevant to what the tool aims to achieve. |
| Living in institution | Literature | Not practical to identify. |
| Length of hospitalisation (1–7 days compared with 8–30 days or > 30 days) | Literature |  |
| **Blood levels** |  |  |
| Patients with abnormal blood results requiring further intervention including, but not limited to red results for clozapine, high creatine kinsae (CK) for antipsychotics (risk of NMS) and lithium levels higher than suggested normal range. | Existing tools | Clozapine and lithium are captured elsewhere. CK was left as a separate indicator. |
| **Hospital related risk indicators** | | |
| **Administration issues** | **Origin** | **Justification** |
| Preparation of the medication by nurses immediately before administration in the care setting (compared with preparing medications at night for the following day) | Literature | It was agreed to exclude these in the stakeholders meeting as they are not practical and will be challenging to measure. |
| Administering nurse being required to carry out other duties during the medication round | Literature |  |
| Number of ‘when required’ doses given at the medication administration round time | Literature |  |
| Number of patients on the ward at the medication administration round time | Literature |  |
| Number of regular doses to be administered in the medication administration round | Literature |  |
| **Pharmacy issues** |  |  |
| Medication reconciliation completed by non pharmacist | Existing tools | This has been discussed with stakeholders, and it was agreed that medication reconciliation is usually completed by pharmacy technicians and seldom by non-pharmacy professionals. |
| Patients requiring medication reconciliation (including return transfers from acute hospitals) | Existing tools | These are normally done by process. |
| Medicines reconciliation issues | Existing tools |  |
| Unresolved medicines reconciliation | Existing tools |  |
| Outstanding clinical queries | Existing tools |  |
| Significant intervention outstanding on handover | Existing tools |  |
| Minor interventions outstanding on handover | Existing tools |  |
| Non-urgent clinical queries - SGA depot requests, medication histories, high anticholinergic burden | Existing tools |  |
| Pharmacy issues requiring follow-up | Existing tools |  |
| Patients with new pharmacy issues identified by the ward team | Existing tools |  |
| **Compliance support** |  |  |
| Compliance support: Yes/No & type. | Existing tools | Repetition, not practical to type. |
| **Discharge issues** |  |  |
| Patients at risk of poor adherence following discharge. | Existing tools | Difficult to identify, and should be cauptured in non-adherance. |
| Discharge patient who are taking ‘high risk’ medicines | Existing tools | This should be captured in high risk medicines. |
| Discharges/Leaves planned within next 72 hours for patients requiring compliance aids. | Existing tools | Compliance captured before, discharge as below. |
| Discharge patients | Existing tools | It was argued that discharge is not a high risk indicator, and it should be discussed in the huddles anyways. Yet, it was kept for the experts to decide as this was used in several current prioritisation tools. These were combined into one and repititions were excluded. |
| Patients being imminently discharged | Existing tools |  |
| Discharge issue resolution by next working day e.g. counselling, MCD, MAR | Existing tools |  |
| Discharges/Leaves planned within next 48 hours | Existing tools |  |
| Discharges/Leaves planned within next 7 days | Existing tools |  |
| **Change of treatment plan** |  |  |
| Sudden/abrupt cessation of medication *which has been agreed as part of a treatment plan* | Existing tools | This was kept, only the agreement as part of treatment plan was removed as it is not practical. |
| Changes to concomitant medicines when on: (Theophylline, Tacrolimus, Ciclosporin, Lithium, Warfarin, Antiretrovirals, Phenytoin, Carbamazepine | Existing tools | These medications should be captured in high risk medicines. |
| **Prescription type** |  | It was agreed to exclude these in the stakeholders meeting as the prescription type is not practical and does not support what the tool should do. |
| Electronic discharge pro forma | Literature |  |
| Prescriptions at admission | Literature |  |
| Discharge prescriptions | Literature |  |
| Re-written items | Literature |  |
| **Type of prescriber** |  |  |
| General practitioner speciality trainee prescribers | Literature | It was agreed to exclude these in the stakeholders meeting as it is not practical. |
| Core medical trainee prescribers | Literature |  |
| Specialty trainee prescriber | Literature |  |
| Staff grade psychiatrist prescriber | Literature |  |
| Consultant prescribers | Literature |  |
| Unknown/locum prescribers | Literature |  |
| **Prescribing process** |  |  |
| Other prescribing issues on medicines administration charts | Existing tools | These are normally done by process. |
| Prescribed medicines by homecare / clinical Trial / secondary care specialist teams | Existing tools | These were reorganised into: Prescribed medicines by homecare  Prescribed medicines by clinical trial Unlicensed medicines Non-formulary medication |
| Unlicensed medicines / off-label / non-formulary prescribing including requests for authorisation | Existing tools |  |
| Supply issue e.g. non-formulary and ULM use | Existing tools |  |
| Supply issues (once investigated) e.g. New non-stock item, multiple missed doses | Existing tools |  |
| Unresolved supply issue e.g. non-formulary and unlicensed medication (ULM) use | Existing tools |  |
| free text drugs | Existing tools |  |
| **New meds** |  |  |
| Unverified newly started medication (non-critical medicines) | Existing tools | These were collated. |
| Multiple new medications for new/ acute medical condition requiring monitoring/ education | Existing tools |  |
| Unverified prescriptions/ Unverified prescriptions excluding suspensions | Existing tools |  |
| **Controlled drugs/critical meds incidents** |  |  |
| on-call/Datix involving critical medicines | Existing tools | The aim of the tool is to prevent DRPs, if an incident happens it should be dealt with immediately. |
| Controlled drugs incidents/Datix including illicit drugs | Existing tools |  |
| **Number of medications** |  |  |
| Polypharmacy | Existing tools | These were collated. |
| Polypharmacy ≥ 10 regular medications with complex regimen e.g. drug-drug or drug-disease interactions, non-compliance with evidence based guidelines. | Existing tools |  |
| Polypharmacy ≥ 10 regular medications in absence of complex regimen and compliant with evidence based guidelines. " | Existing tools |  |
| Problematic polypharmacy | Existing tools |  |
| Count of unverified prescriptions | Existing tools |  |
| Count of prescriptions under review | Existing tools |  |
| Increased number of medications (>5) | Literature |  |
| Increased number of medications (>6) | Literature |  |
| Increased number of medications (>11) | Literature |  |
| Potentially inappropriate medications (>1) | Literature | Not practical, we need to review the patient to identify potentially inappropriate medications. |
| Potentially inappropriate medications (>2) | Literature |  |

**Appendix.3**

**Modified and added risk indicators for Delphi study one**

| **Original risk indicator** | **Modified risk indicator** | **Justification** |
| --- | --- | --- |
| Antimicrobials or antivirals (e.g. vancomycin, itraconazole) | Antimicrobials or antivirals (e.g. amoxicillin, nystatin) | One commented (Rarely treated in acute mental health sites as infections are commonly treated in this setting – could this be misleading?)  This comment might have originated due to the examples of antimicrobials provided (vancomycin and itraconazole) which are generally used in acute hospitals. Changing these examples to more commonly used items like amoxicillin and nystatin could clarify this. |
| Anticonvulsants (e.g. topiramate, levetriacetam) | Anticonvulsants (e.g. topiramate, levetriacetam) for epilepsy | Participants rated it differently based on the indication, so it was modified to focus on epilepsy as mood stabilisers are listed separately. |
| Anticoagulants | Prescribed direct oral anticoagulant (DOAC) medication | To avoid confusion as warfarin is listed as a separate risk indicator. |
| Female of child bearing potential prescribed sodium valproate | Female of child bearing potential prescribed teratogenic medicines such as sodium valproate | Pregnancy was suggested as a risk indicator, this would cover the risk by including other teratogenic medicines. |
| Patients > 65 years (age) | Patients > 70 years (age) | There was no agreement on this risk indicator, and one participants suggested age >70 |
| Patient requires rapid-tranquillisation | Patient requires oral ‘when required’ psychotropic for agitation | Some participants did not understand how it differs from IM rapid tranquillisation. |
| Patients who did not have VTE (Venous thromboembolism) assessment | No VTE assessment for those prescribed antipsychotic. | There was no agreement on this statement so it was modified as some participants commented that the risk is associated with antipsychotics use. |
| T2/T3 renewal needed | Patient prescribed medication prompting review of T2/T3* | To clarify the statement. |

**Added risk indicators:**

1. Patients < 12 years (age)
2. Patients < 18 years (age)
3. Patients > 80 years (age)
4. More than one regular antidepressant prescribed
5. Patients taking laxatives/ have constipation
6. Patients who have recently stopped or started smoking
7. Patients recently moved from another country (difficult to obtain history, different medications brands)
8. Patients who self harm or have suicidal thoughts
9. Patients with non-adherence
10. Patient with dementia or cognitive impairment prescribed one or more antimuscarinics
11. Missed doses of high risk medications
12. Missed doses of high risk mental health medications
13. Low sodium levels in a patient taking one or more antidepressants
14. Patients prescribed off-label medicines

**Appendix.4**

**Modified and added risk indicators for Delphi study two**

| **Original risk indicator** | **Modified risk indicator** | **Justification** |
| --- | --- | --- |
| Individual patients identified as ‘medium risk’ following completion of the prioritisation tool should be reviewed by or discussed with a senior pharmacist. | Individual patients identified as ‘medium risk’ following completion of the prioritisation tool should be reviewed by or discussed with an experienced pharmacists in the relevant area. | The term ‘senior’ was modified to ‘experienced pharmacists in the relevant area’ as per panel members’ feedback. |
| Individual patients identified as ‘high-risk’ following completion of the prioritisation tool should be reviewed by or discussed with a senior pharmacist. | Individual patients identified as ‘high-risk’ following completion of the prioritisation tool should be reviewed by or discussed with an experienced pharmacists in the relevant area. | The term ‘senior’ was modified to ‘experienced pharmacists in the relevant area’ as per panel members’ feedback. |

**Added statement:**

1. For high-risk, we added ‘a statement for 3 times a week’.

**Changes to the tool:**

- 1. Removed the code and added tick boxes for pharmacist/pharmacy technician.
  2. Removed the signature.
  3. Removed the admission and assessment time.
  4. Modified the instructions by adding the purpose of the tool and explaining some possible uses for the comment section.
  5. Modified the instructions about the comment – changed ‘outcomes’ to ‘actions’.

**Appendix.5**

**Table 1. Results of round 1 of Delphi study 1.**

|  | **Risk Indicators** | | **Risk^#^** | | | | | | **Importance^##^** | | | | | |
| --- | --- | --- | --- | --- | --- | --- | --- | --- | --- | --- | --- | --- | --- | --- |
|  |  |  | **N** | **Median** | **Mean** | **1-2%** | **3-5%** | **6-7%** | **N** | **Median** | **Mean** | **1-4%** | **5-7%** | **6-7%** |
| 1 | Clozapine | | 36 | 7 | 6.7 | 0.0 | 2.8 | 97.2 | 36 | 7 | 6.8 | 0.0 | 100.0 | 100.0 |
| 2 | Lithium | | 36 | 7 | 6.6 | 0.0 | 2.8 | 97.2 | 36 | 7 | 6.7 | 0.0 | 100.0 | 97.2 |
| 3 | Insulin | | 35 | 7 | 6.5 | 0.0 | 5.7 | 94.3 | 35 | 7 | 6.5 | 0.0 | 100.0 | 88.6 |
| 4 | Significant^a^ drug interaction | | 36 | 6 | 6.4 | 0.0 | 5.6 | 94.4 | 36 | 6 | 6.3 | 0.0 | 100.0 | 86.1 |
| 5 | Missed mental health medications | | 36 | 5.5 | 5.7 | 0.0 | 50.0 | 50.0 | 36 | 6 | 6.0 | 0.0 | 100.0 | 66.7 |
| 6 | Presence of a significant^b^ adverse drug reaction | | 36 | 7 | 6.5 | 0.0 | 11.1 | 88.9 | 36 | 7 | 6.4 | 2.8 | 97.2 | 86.1 |
| 7 | More than one regular antipsychotic prescribed | | 36 | 6 | 5.9 | 0.0 | 22.2 | 77.8 | 36 | 6 | 6.1 | 2.8 | 97.2 | 88.9 |
| 8 | High Dose Antipsychotic Therapy (above 100% BNF maximum) prescribed | | 36 | 6 | 6.3 | 0.0 | 2.8 | 97.2 | 36 | 6 | 6.3 | 2.8 | 97.2 | 86.1 |
| 9 | Female of child bearing potential prescribed sodium valproate | | 36 | 7 | 6.8 | 0.0 | 0.0 | 100.0 | 36 | 7 | 6.7 | 2.8 | 97.2 | 97.2 |
| 10 | Prescribed a QTc prolonging medication (e.g chlorpromazine, quetiapine, amisulpride) | | 36 | 6 | 5.9 | 0.0 | 25.0 | 75.0 | 36 | 6 | 5.9 | 2.8 | 97.2 | 75.0 |
| 11 | Patient requires intramuscular rapid-tranquillisation administration | | 36 | 6 | 6.3 | 0.0 | 11.1 | 88.9 | 36 | 6.5 | 6.3 | 2.8 | 97.2 | 86.1 |
| 12 | Toxic clozapine serum levels | | 36 | 7 | 6.8 | 0.0 | 0.0 | 100.0 | 36 | 7 | 6.6 | 2.8 | 97.2 | 94.4 |
| 13 | Toxic lithium blood levels | | 36 | 7 | 6.9 | 0.0 | 0.0 | 100.0 | 36 | 7 | 6.8 | 2.8 | 97.2 | 94.4 |
| 14 | QTc results outside reference range | | 36 | 6 | 6.3 | 0.0 | 11.1 | 88.9 | 36 | 6 | 6.1 | 2.8 | 97.2 | 80.6 |
| 15 | Antipsychotics (e.g. risperidone, haloperidol) | | 36 | 6 | 5.4 | 2.8 | 41.7 | 55.6 | 36 | 6 | 6.0 | 5.6 | 94.4 | 72.2 |
| 16 | Strong opioids (e.g. methadone, fentanyl) | | 36 | 6.5 | 6.4 | 0.0 | 13.9 | 86.1 | 36 | 6 | 6.0 | 5.6 | 94.4 | 77.8 |
| 17 | Missed doses | | 36 | 5.5 | 5.5 | 0.0 | 50.0 | 50.0 | 36 | 6 | 5.7 | 5.6 | 94.4 | 52.8 |
| 18 | Patient requires rapid-tranquillisation | | 36 | 6 | 5.9 | 0.0 | 33.3 | 66.7 | 36 | 6 | 5.9 | 5.6 | 94.4 | 63.9 |
| 19 | Patients regularly spitting out or refusing medication | | 36 | 6 | 5.6 | 2.8 | 36.1 | 61.1 | 36 | 6 | 5.8 | 5.6 | 94.4 | 61.1 |
| 20 | Zuclopenthixol acetate or Zuclopentixol Acuphase | | 36 | 6.5 | 6.4 | 0.0 | 8.3 | 91.7 | 36 | 7 | 6.3 | 8.3 | 91.7 | 86.1 |
| 21 | Valproate | | 36 | 6 | 6.3 | 0.0 | 16.7 | 83.3 | 36 | 6 | 6.2 | 8.3 | 91.7 | 86.1 |
| 22 | Any single drug above British National Formulary limits (unless planned detoxification) | | 36 | 6 | 6.0 | 0.0 | 19.4 | 80.6 | 36 | 6 | 5.9 | 8.3 | 91.7 | 66.7 |
| 23 | Patients with physical healthcare issues requiring follow-up | | 36 | 6 | 5.6 | 0.0 | 44.4 | 55.6 | 36 | 5 | 5.4 | 8.3 | 91.7 | 41.7 |
| 24 | Polypharmacy ≥ 10 regular medications | | 36 | 6 | 6.3 | 0.0 | 13.9 | 86.1 | 36 | 6 | 6.1 | 8.3 | 91.7 | 77.8 |
| 25 | Sudden/abrupt cessation of medication | | 36 | 6 | 5.7 | 0.0 | 36.1 | 63.9 | 36 | 6 | 5.7 | 8.3 | 91.7 | 58.3 |
| 26 | New T2/T3 under the Mental Health Act | | 36 | 5 | 5.1 | 5.6 | 58.3 | 36.1 | 36 | 6 | 5.6 | 8.3 | 91.7 | 55.6 |
| 27 | Depot antipsychotics | | 36 | 6 | 5.5 | 0.0 | 47.2 | 52.8 | 36 | 6 | 5.6 | 11.1 | 88.9 | 58.3 |
| 28 | More than one hypnotic prescribed | | 36 | 6 | 5.6 | 2.8 | 44.4 | 52.8 | 36 | 6 | 5.5 | 11.1 | 88.9 | 61.1 |
| 29 | Increase of a regular psychotropic within 7 days of the last increase (unless as part of a dose titration regimen) | | 36 | 5 | 5.4 | 0.0 | 52.8 | 47.2 | 36 | 5.5 | 5.4 | 11.1 | 88.9 | 50.0 |
| 30 | Acute renal impairment | | 36 | 6.5 | 6.4 | 0.0 | 8.3 | 91.7 | 36 | 6 | 6.0 | 11.1 | 88.9 | 83.3 |
| 31 | Patients receiving electro-convulsive therapy^c^ | | 36 | 6 | 5.8 | 0.0 | 27.8 | 72.2 | 36 | 6 | 5.7 | 11.1 | 88.9 | 61.1 |
| 32 | Electrolytes levels outside reference range | | 36 | 6 | 5.6 | 0.0 | 44.4 | 55.6 | 36 | 5 | 5.4 | 11.1 | 88.9 | 44.4 |
| 33 | Outstanding Electrocardiogram (ECG) | | 36 | 5 | 5.5 | 0.0 | 52.8 | 47.2 | 36 | 5.5 | 5.6 | 11.1 | 88.9 | 50.0 |
| 34 | Patients with unverified newly started medication | | 36 | 6 | 5.6 | 0.0 | 41.7 | 58.3 | 36 | 6 | 5.7 | 11.1 | 88.9 | 63.9 |
| 35 | Chronic kidney Disease Stage > 4 (eGFR of 15 to 29ml/min) | | 35 | 7 | 6.7 | 0.0 | 0.0 | 100.0 | 34 | 7 | 6.3 | 11.8 | 88.2 | 88.2 |
| 36 | Chronic kidney Disease Stage > 5 (eGFR below 15ml/min) | | 34 | 7 | 6.9 | 0.0 | 0.0 | 100.0 | 33 | 7 | 6.4 | 12.1 | 87.9 | 87.9 |
| 37 | Anticonvulsants (e.g. topiramate, levetriacetam) | | 36 | 6 | 5.9 | 0.0 | 25.0 | 75.0 | 36 | 6 | 5.8 | 13.9 | 86.1 | 66.7 |
| 38 | Anticoagulants | | 36 | 6 | 5.8 | 0.0 | 27.8 | 72.2 | 36 | 6 | 5.5 | 13.9 | 86.1 | 52.8 |
| 39 | Chronic kidney Disease Stage > 3a (eGFR of 45 to 59ml/min) | | 36 | 6 | 5.9 | 0.0 | 30.6 | 69.4 | 36 | 6 | 5.5 | 13.9 | 86.1 | 61.1 |
| 40 | Patients with substance abuse | | 36 | 6 | 5.6 | 0.0 | 47.2 | 52.8 | 36 | 5 | 5.4 | 13.9 | 86.1 | 41.7 |
| 41 | Patients planned for discharge/leave | | 36 | 5 | 4.8 | 11.1 | 52.8 | 36.1 | 36 | 6 | 5.6 | 13.9 | 86.1 | 63.9 |
| 42 | Polypharmacy ≥ 5 regular medications | | 36 | 6 | 5.6 | 0.0 | 41.7 | 58.3 | 36 | 5 | 5.4 | 13.9 | 86.1 | 44.4 |
| 43 | Chronic kidney Disease Stage > 3b (eGFR of 30 to 44ml/min) | | 36 | 6 | 6.3 | 0.0 | 16.7 | 83.3 | 35 | 6 | 5.9 | 14.3 | 85.7 | 77.1 |
| 44 | Fall > 1 in the preceding 3 months | | 35 | 6 | 5.6 | 0.0 | 48.6 | 51.4 | 35 | 5 | 5.4 | 14.3 | 85.7 | 40.0 |
| 45 | Patients with new swallowing difficulties | | 35 | 6 | 5.9 | 0.0 | 20.0 | 80.0 | 35 | 6 | 5.5 | 14.3 | 85.7 | 60.0 |
| 46 | Patients in seclusion | | 35 | 6 | 5.9 | 0.0 | 31.4 | 68.6 | 35 | 6 | 5.6 | 14.3 | 85.7 | 54.3 |
| 47 | Patients with swallowing difficulties/ Nil by mouth | | 34 | 6 | 5.9 | 2.9 | 23.5 | 73.5 | 34 | 6 | 5.8 | 14.7 | 85.3 | 67.6 |
| 48 | Medications for Parkinson's disease (e.g. levodopa, apomorphine) | | 36 | 6 | 6.0 | 0.0 | 25.0 | 75.0 | 36 | 6 | 5.4 | 16.7 | 83.3 | 52.8 |
| 49 | Alcohol detox medications (Pabrinex and/or Chlordiazepoxide) | | 36 | 6 | 5.6 | 5.6 | 27.8 | 66.7 | 36 | 6 | 5.6 | 16.7 | 83.3 | 63.9 |
| 50 | More than one anxiolytic prescribed | | 36 | 5 | 5.4 | 2.8 | 50.0 | 47.2 | 36 | 5 | 5.3 | 16.7 | 83.3 | 44.4 |
| 51 | More than two mood stabilisers prescribed | | 36 | 5 | 5.4 | 0.0 | 52.8 | 47.2 | 36 | 6 | 5.5 | 16.7 | 83.3 | 52.8 |
| 52 | Patients receiving covert medications | | 36 | 6 | 5.5 | 0.0 | 36.1 | 63.9 | 36 | 6 | 5.6 | 16.7 | 83.3 | 58.3 |
| 53 | White blood cells (WBC) levels outside reference range | | 36 | 6 | 5.8 | 0.0 | 44.4 | 55.6 | 36 | 5.5 | 5.4 | 16.7 | 83.3 | 50.0 |
| 54 | Patient not reviewed within the past 7 days (acute) or fortnight (rehab) by a pharmacist | | 36 | 5 | 5.4 | 0.0 | 55.6 | 44.4 | 36 | 6 | 5.6 | 16.7 | 83.3 | 63.9 |
| 55 | T2/T3 renewal needed under the Mental Health Act | | 36 | 5 | 4.9 | 8.3 | 55.6 | 36.1 | 36 | 6 | 5.4 | 16.7 | 83.3 | 52.8 |
| 56 | Patients with high creatine kinase (CK) | | 35 | 6 | 6.0 | 0.0 | 37.1 | 62.9 | 35 | 6 | 5.5 | 17.1 | 82.9 | 51.4 |
| 57 | Acute hepatic impairment (Liver function tests > 3 times upper of limit normal) | | 34 | 6 | 6.3 | 0.0 | 14.7 | 85.3 | 34 | 6 | 5.7 | 17.6 | 82.4 | 67.6 |
| 58 | Patients with Behavioural and Psychological Symptoms of Dementia (BPSD) when not on a dementia ward | | 34 | 6 | 5.8 | 0.0 | 32.4 | 67.6 | 33 | 5 | 5.3 | 18.2 | 81.8 | 48.5 |
| 59 | Patients prescribed medicines as part of a clinical trial | | 33 | 6 | 5.7 | 0.0 | 30.3 | 69.7 | 33 | 6 | 5.5 | 18.2 | 81.8 | 63.6 |
| 60 | Warfarin | | 35 | 7 | 6.6 | 0.0 | 11.4 | 88.6 | 36 | 6 | 5.6 | 19.4 | 80.6 | 55.6 |
| 61 | Chronic hepatic impairment | | 36 | 6 | 5.9 | 0.0 | 19.4 | 80.6 | 36 | 6 | 5.4 | 19.4 | 80.6 | 66.7 |
| 62 | Patients lacking capacity to consent to medication administration | | 36 | 5 | 5.1 | 5.6 | 61.1 | 33.3 | 36 | 5 | 5.2 | 19.4 | 80.6 | 33.3 |
| 63 | Patients prescribed unlicensed medicines | | 36 | 5 | 5.4 | 2.8 | 52.8 | 44.4 | 36 | 5 | 5.3 | 19.4 | 80.6 | 41.7 |
| 64 | Formulation review required e.g. NG, PEG, JEJ | | 32 | 6 | 6.1 | 0.0 | 25.0 | 75.0 | 32 | 6 | 5.4 | 25.0 | 75.0 | 59.4 |
| 65 | Patients on the palliative care pathway. | | 32 | 6 | 5.9 | 0.0 | 37.5 | 62.5 | 32 | 6 | 5.4 | 25.0 | 75.0 | 59.4 |
| 66 | Patients > 65 years (age) | | 36 | 5 | 5.2 | 0.0 | 58.3 | 41.7 | 36 | 5 | 5.1 | 25.0 | 75.0 | 41.7 |
| 67 | Patients with undetermined allergy status | | 36 | 6 | 5.7 | 5.6 | 25.0 | 69.4 | 36 | 6 | 5.3 | 25.0 | 75.0 | 52.8 |
| 68 | Patients with new compliance aid requested | | 36 | 5 | 4.9 | 2.8 | 55.6 | 41.7 | 36 | 5 | 5.1 | 25.0 | 75.0 | 41.7 |
| 69 | Intensive Therapeutic Drug Monitoring (TDM) drugs (e.g. phenytoin, carbamazepine) | | 34 | 6.5 | 6.3 | 0.0 | 20.6 | 79.4 | 34 | 6 | 5.5 | 26.5 | 73.5 | 64.7 |
| 70 | Patients who did not have VTE (Venous thromboembolism) assessment | | 35 | 6 | 5.5 | 5.7 | 34.3 | 60.0 | 35 | 5 | 5.1 | 28.6 | 71.4 | 48.6 |
| 71 | Haemoglobin levels (CRP, HB1) outside reference range | | 32 | 5 | 5.2 | 0.0 | 65.6 | 34.4 | 31 | 5 | 4.7 | 29.0 | 71.0 | 22.6 |
| 72 | Moderate hepatic impairment (Liver function tests > upper limit of normal (ULN) but < 3X ULN) | | 34 | 6 | 5.6 | 2.9 | 32.4 | 64.7 | 34 | 6 | 5.1 | 29.4 | 70.6 | 58.8 |
| 73 | Antimuscarinics (e.g. procyclidine, oxybutynin) | | 36 | 5 | 4.8 | 5.6 | 66.7 | 27.8 | 36 | 5 | 5.1 | 30.6 | 69.4 | 44.4 |
| 74 | Chronic kidney Disease Stage > 2 (slightly reduced eGFR of 60 to 89ml/min, with other signs of kidney damage) | | 36 | 5 | 5.1 | 5.6 | 52.8 | 41.7 | 36 | 5 | 4.8 | 30.6 | 69.4 | 36.1 |
| 75 | Patients prescribed non-formulary medication | | 36 | 5 | 4.9 | 0.0 | 72.2 | 27.8 | 36 | 5 | 5.0 | 30.6 | 69.4 | 38.9 |
| 76 | Anti-cancer medications (e.g. azathioprine, fluorouracil) | | 32 | 6.5 | 6.3 | 0.0 | 9.4 | 90.6 | 32 | 5 | 4.8 | 31.3 | 68.8 | 43.8 |
| 77 | Phenytoin | | 36 | 6 | 5.8 | 2.8 | 27.8 | 69.4 | 36 | 5.5 | 4.9 | 33.3 | 66.7 | 50.0 |
| 78 | Hospitalisation due to psychiatric condition > 1 in the preceding year | | 36 | 5 | 5.0 | 5.6 | 61.1 | 33.3 | 36 | 5 | 4.9 | 33.3 | 66.7 | 33.3 |
| 79 | Patients on self-administration | | 36 | 5 | 4.5 | 11.1 | 66.7 | 22.2 | 36 | 5 | 4.8 | 38.9 | 61.1 | 30.6 |
| 80 | Medicines compliance aid from community pharmacy e.g dosette, pillpouch | | 36 | 5 | 4.4 | 5.6 | 77.8 | 16.7 | 36 | 5 | 4.6 | 38.9 | 61.1 | 27.8 |
| 81 | Chronic kidney Disease Stage > 1 (normal eGFR above 90ml/min, but other tests have detected signs of kidney damage) | | 35 | 5 | 4.6 | 14.3 | 57.1 | 28.6 | 35 | 5 | 4.5 | 40.0 | 60.0 | 31.4 |
| 82 | Patient not spoken to by a pharmacy member within the past 7 days (acute) or fortnight (rehab) | | 36 | 5 | 4.4 | 8.3 | 66.7 | 25.0 | 36 | 5 | 4.5 | 41.7 | 58.3 | 27.8 |
| 83 | Antimicrobials or antivirals (e.g. vancomycin, itraconazole) | | 35 | 6 | 5.6 | 0.0 | 34.3 | 65.7 | 35 | 5 | 4.4 | 42.9 | 57.1 | 34.3 |
| 84 | Patients prescribed medicines by homecare | | 30 | 5 | 4.5 | 13.3 | 63.3 | 23.3 | 30 | 5 | 4.4 | 43.3 | 56.7 | 23.3 |
| 85 | Venlafaxine | | 36 | 5 | 4.5 | 5.6 | 77.8 | 16.7 | 36 | 5 | 4.4 | 44.4 | 55.6 | 16.7 |
| 86 | Short course of Steroids | | 36 | 5 | 5.1 | 2.8 | 55.6 | 41.7 | 36 | 5 | 4.2 | 44.4 | 55.6 | 16.7 |
| 87 | Patient taking anti-dementia medications | | 36 | 4 | 4.2 | 11.1 | 72.2 | 16.7 | 36 | 5 | 4.6 | 44.4 | 55.6 | 27.8 |
| 88 | Digoxin | | 35 | 6 | 5.8 | 0.0 | 37.1 | 62.9 | 35 | 5 | 4.5 | 45.7 | 54.3 | 25.7 |
| 89 | Patients diagnosed with COVID | | 34 | 5 | 5.3 | 5.9 | 50.0 | 44.1 | 34 | 5 | 4.4 | 47.1 | 52.9 | 29.4 |
| 90 | Paroxetine | | 36 | 5 | 4.6 | 5.6 | 75.0 | 19.4 | 36 | 5 | 4.4 | 47.2 | 52.8 | 19.4 |
| 91 | Patient taking antidepressants | | 36 | 4 | 4.2 | 11.1 | 69.4 | 19.4 | 36 | 5 | 4.6 | 47.2 | 52.8 | 33.3 |
| 92 | Contraceptives (e.g. Yasmin, Evra) | | 35 | 4 | 4.1 | 17.1 | 74.3 | 8.6 | 35 | 5 | 4.2 | 48.6 | 51.4 | 20.0 |
| 93 | Amiodarone | | 34 | 6 | 5.6 | 0.0 | 47.1 | 52.9 | 34 | 4 | 4.2 | 52.9 | 47.1 | 23.5 |
| 94 | Hydrocortisone tablets (for adrenal insufficiency/Addison's disease) | | 35 | 6 | 5.6 | 0.0 | 42.9 | 57.1 | 35 | 4 | 4.5 | 54.3 | 45.7 | 34.3 |
| 95 | Aminophylline | | 32 | 5.5 | 5.4 | 3.1 | 46.9 | 50.0 | 33 | 4 | 4.0 | 54.5 | 45.5 | 27.3 |
| 96 | Desmopressin (for cranial diabetes insipidus) | | 30 | 5 | 5.0 | 3.3 | 53.3 | 43.3 | 31 | 4 | 4.1 | 54.8 | 45.2 | 29.0 |
| 97 | Type I, III or IV antiarrhythmics | | 30 | 5 | 5.2 | 3.3 | 53.3 | 43.3 | 29 | 4 | 4.0 | 55.2 | 44.8 | 24.1 |
| 98 | Theophylline | | 34 | 6 | 5.6 | 0.0 | 47.1 | 52.9 | 34 | 4 | 3.9 | 55.9 | 44.1 | 20.6 |
| 99 | Patient with daily aseptic needs e.g. on Total Parenteral Nutrition, antibiotic infusion | | 26 | 6 | 6.2 | 0.0 | 19.2 | 80.8 | 25 | 4 | 4.0 | 56.0 | 44.0 | 32.0 |
| 100 | Hospitalisation due to non-psychiatric condition > 1 in the preceding year | | 35 | 5 | 4.7 | 5.7 | 62.9 | 31.4 | 35 | 4 | 4.2 | 60.0 | 40.0 | 14.3 |
| 101 | Digoxin, amiodarone loading | | 29 | 6 | 6.2 | 0.0 | 20.7 | 79.3 | 28 | 3.5 | 3.9 | 60.7 | 39.3 | 32.1 |
| 102 | Diuretics | | 36 | 5 | 4.4 | 5.6 | 77.8 | 16.7 | 36 | 4 | 3.9 | 66.7 | 33.3 | 8.3 |
| 103 | Tretinoin | | 29 | 5 | 5.2 | 6.9 | 51.7 | 41.4 | 30 | 3.5 | 3.5 | 66.7 | 33.3 | 10.0 |
| 104 | Angiotensin Converting Enzyme Inhibitors (ACEIs) or Angiotensin Receptor Inhibitors (ARBs) | | 36 | 4 | 4.0 | 13.9 | 80.6 | 5.6 | 36 | 4 | 3.7 | 69.4 | 30.6 | 5.6 |
| 105 | Magnesium supplements (e.g. magnesium glycinate, magnesium aspartate) | | 33 | 4 | 4.0 | 15.2 | 75.8 | 9.1 | 33 | 4 | 3.8 | 72.7 | 27.3 | 6.1 |
| 106 | Diltiazem | | 34 | 5 | 4.4 | 8.8 | 70.6 | 20.6 | 34 | 3 | 3.5 | 73.5 | 26.5 | 5.9 |
| 107 | Hormone Replacement Therapy (HRT) (e.g. Premique, Premarin) | | 36 | 3.5 | 3.6 | 27.8 | 63.9 | 8.3 | 36 | 3 | 3.1 | 75.0 | 25.0 | 5.6 |
| 108 | Selective Estrogen Receptor Modulators (SERMs) (e.g. tamoxifen, raloxifene) | | 33 | 4 | 4.5 | 6.1 | 63.6 | 30.3 | 33 | 3 | 3.4 | 78.8 | 21.2 | 12.1 |
| 109 | Bisphosphonates (e.g. alendronate, risedronate) | | 35 | 4 | 3.9 | 20.0 | 68.6 | 11.4 | 35 | 3 | 3.3 | 82.9 | 17.1 | 5.7 |
|  | | **^#^**Low-risk if >75% rated 1-2, medium-risk if >75% rated 3-5, and high-risk if >75% rated 6-7. **^##^**Included in the tool if >75% rated importance 6-7 or >85% rated importance 5-7. ^a^ An interaction occurred that either requires action to be taken to avoid harm or may have contributed to or resulted in patient harm. ^b^ An adverse drug reaction occurred that may have contributed to or resulted in patient harm. ^c^ Referring to related medication management issues. PEG: percutaneous endoscopic gastrostomy. NG:nasogastric tube. JEJ:jejunostomy tube. The red line indicate the end of included risk indicators and start of excluded risk indicators. | | | | | | | | | | | | |

**Table 2. Results of round 2 of Delphi study 1.**

|  | **Risk Indicators** | | **Risk^#^** | | | | | | **Importance^##^** | | | | | |  |  |
| --- | --- | --- | --- | --- | --- | --- | --- | --- | --- | --- | --- | --- | --- | --- | --- | --- |
|  |  |  | **N** | **Median** | **Mean** | **1-2%** | **3-5%** | **6-7%** | **N** | **Median** | **Mean** | **1-4%** | **5-7%** | **6-7%** |  |  |
| 1 | Antipsychotics (e.g. risperidone, haloperidol) | | 29 | 6 | 5.6 | 0.0 | 31.0 | 69.0 | 29 | 6 | 5.9 | 0.0 | 100.0 | 75.9 |  |  |
| 2 | Anticonvulsants (e.g. topiramate, levetriacetam) for epilepsy | | 29 | 6 | 6.0 | 0.0 | 17.2 | 82.8 | 29 | 6 | 5.9 | 0.0 | 100.0 | 75.9 |  |  |
| 3 | Strong opioids (e.g. methadone, fentanyl) | | 29 | 6 | 6.3 | 0.0 | 6.9 | 93.1 | 29 | 6 | 6.0 | 0.0 | 100.0 | 82.8 |  |  |
| 4 | Clozapine | | 29 | 7 | 6.9 | 0.0 | 0.0 | 100 | 29 | 7 | 6.9 | 0.0 | 100.0 | 100.0 |  |  |
| 5 | Lithium | | 29 | 7 | 6.8 | 0.0 | 0.0 | 100 | 29 | 7 | 6.9 | 0.0 | 100.0 | 100.0 |  |  |
| 6 | Insulin | | 28 | 7 | 6.8 | 0.0 | 0.0 | 100 | 28 | 7 | 6.8 | 0.0 | 100.0 | 100.0 |  |  |
| 7 | Valproate | | 29 | 6 | 6.3 | 0.0 | 6.9 | 93.1 | 29 | 6 | 6.3 | 0.0 | 100.0 | 89.7 |  |  |
| 8 | Missed mental health medications | | 29 | 5 | 5.4 | 0.0 | 51.7 | 48.3 | 29 | 6 | 5.7 | 0.0 | 100.0 | 65.5 |  |  |
| 9 | Chronic kidney Disease Stage > 3a (eGFR of 45 to 59ml/min) | | 29 | 6 | 5.9 | 0.0 | 27.6 | 72.4 | 29 | 6 | 5.8 | 0.0 | 100.0 | 75.9 |  |  |
| 10 | Chronic kidney Disease Stage > 3b (eGFR of 30 to 44ml/min) | | 29 | 6 | 6.2 | 0.0 | 13.8 | 86.2 | 29 | 6 | 6.0 | 0.0 | 100.0 | 86.2 |  |  |
| 11 | Chronic kidney Disease Stage > 4 (eGFR of 15 to 29ml/min) | | 29 | 7 | 6.9 | 0.0 | 0.0 | 100 | 29 | 7 | 6.9 | 0.0 | 100.0 | 100.0 |  |  |
| 12 | Chronic kidney Disease Stage > 5 (eGFR below 15ml/min) | | 29 | 7 | 7.0 | 0.0 | 0.0 | 100 | 28 | 7 | 6.9 | 0.0 | 100.0 | 100.0 |  |  |
| 13 | Patients with new swallowing difficulties | | 28 | 6 | 5.9 | 0.0 | 17.9 | 82.1 | 28 | 6 | 5.8 | 0.0 | 100.0 | 75.0 |  |  |
| 14 | Formulation review required e.g. NG, PEG, JEJ | | 26 | 6 | 6.0 | 0.0 | 11.5 | 88.5 | 26 | 6 | 5.8 | 0.0 | 100.0 | 80.8 |  |  |
| 15 | Patients with Behavioural and Psychological Symptoms of Dementia (BPSD) when not on a dementia ward | | 27 | 6 | 5.8 | 0.0 | 29.6 | 70.4 | 27 | 5 | 5.6 | 0.0 | 100.0 | 44.4 |  |  |
| 16 | Patients receiving covert medications | | 29 | 6 | 5.8 | 0.0 | 24.1 | 75.9 | 29 | 6 | 5.9 | 0.0 | 100.0 | 75.9 |  |  |
| 17 | Patients regularly spitting out or refusing medication | | 29 | 6 | 5.7 | 3.4 | 27.6 | 69.0 | 29 | 6 | 5.8 | 0.0 | 100.0 | 79.3 |  |  |
| 18 | Patients with high creatine kinase (CK) | | 28 | 6 | 5.9 | 0.0 | 28.6 | 71.4 | 28 | 6 | 5.8 | 0.0 | 100.0 | 64.3 |  |  |
| 19 | Toxic clozapine serum levels | | 29 | 7 | 6.9 | 0.0 | 0.0 | 100 | 29 | 7 | 6.9 | 0.0 | 100.0 | 100.0 |  |  |
| 20 | Toxic lithium blood levels | | 29 | 7 | 7.0 | 0.0 | 0.0 | 100 | 29 | 7 | 7.0 | 0.0 | 100.0 | 100.0 |  |  |
| 21 | QTc results outside reference range | | 29 | 6 | 6.2 | 0.0 | 6.9 | 93.1 | 29 | 6 | 6.1 | 0.0 | 100.0 | 93.1 |  |  |
| 22 | Patients prescribed medicines as part of a clinical trial | | 25 | 6 | 5.8 | 0.0 | 16.0 | 84.0 | 25 | 6 | 5.9 | 0.0 | 100.0 | 80.0 |  |  |
| 23 | Patients with unverified newly started medication | | 29 | 6 | 5.7 | 0.0 | 27.6 | 72.4 | 29 | 6 | 5.9 | 0.0 | 100.0 | 75.9 |  |  |
| 24 | Patients prescribed unlicensed medicines | | 29 | 5 | 5.5 | 0.0 | 58.6 | 41.4 | 29 | 5 | 5.4 | 0.0 | 100.0 | 34.5 |  |  |
| 25 | Sudden/abrupt cessation of medication | | 29 | 6 | 5.8 | 0.0 | 24.1 | 75.9 | 29 | 6 | 5.8 | 0.0 | 100.0 | 75.9 |  |  |
| 26 | Depot antipsychotics | | 29 | 6 | 5.7 | 0.0 | 31.0 | 69.0 | 29 | 6 | 5.9 | 3.4 | 96.6 | 75.9 |  |  |
| 27 | Zuclopenthixol acetate or Zuclopentixol Acuphase | | 29 | 7 | 6.6 | 0.0 | 0.0 | 100 | 29 | 7 | 6.5 | 3.4 | 96.6 | 96.6 |  |  |
| 28 | Presence of a significant^a^ adverse drug reaction (ADR) | | 29 | 7 | 6.7 | 0.0 | 3.4 | 96.6 | 29 | 7 | 6.6 | 3.4 | 96.6 | 96.6 |  |  |
| 29 | Significant^b^ drug interaction | | 29 | 6 | 6.2 | 0.0 | 6.9 | 93.1 | 29 | 6 | 6.1 | 3.4 | 96.6 | 86.2 |  |  |
| 30 | More than one regular antipsychotic prescribed | | 29 | 6 | 6.0 | 0.0 | 13.8 | 86.2 | 29 | 6 | 6.0 | 3.4 | 96.6 | 89.7 |  |  |
| 31 | More than one hypnotic prescribed | | 29 | 6 | 5.7 | 0.0 | 31.0 | 69.0 | 29 | 6 | 5.6 | 3.4 | 96.6 | 58.6 |  |  |
| 32 | Patient with dementia or cognitive impairment prescribed one or more antimuscarinics | | 29 | 6 | 6.1 | 0.0 | 17.2 | 82.8 | 29 | 6 | 5.8 | 3.4 | 96.6 | 75.9 |  |  |
| 33 | Missed doses | | 29 | 5 | 5.4 | 0.0 | 51.7 | 48.3 | 29 | 5 | 5.4 | 3.4 | 96.6 | 44.8 |  |  |
| 34 | Missed doses of high-risk medications | | 29 | 6 | 6.4 | 0.0 | 3.4 | 96.6 | 29 | 6 | 6.3 | 3.4 | 96.6 | 93.1 |  |  |
| 35 | Missed doses of high-risk mental health medications | | 29 | 6 | 6.3 | 0.0 | 6.9 | 93.1 | 29 | 6 | 6.3 | 3.4 | 96.6 | 93.1 |  |  |
| 36 | High Dose Antipsychotic Therapy (above 100% BNF maximum) prescribed | | 29 | 6 | 6.1 | 0.0 | 6.9 | 93.1 | 29 | 6 | 6.1 | 3.4 | 96.6 | 93.1 |  |  |
| 37 | Any single drug above BNF limits (unless planned detoxification) | | 29 | 6 | 5.7 | 0.0 | 41.4 | 58.6 | 29 | 6 | 5.6 | 3.4 | 96.6 | 65.5 |  |  |
| 38 | Female of child bearing potential prescribed teratogenic medicines such as sodium valproate | | 29 | 7 | 7.0 | 0.0 | 0.0 | 100 | 29 | 7 | 6.8 | 3.4 | 96.6 | 96.6 |  |  |
| 39 | Patient requires intramuscular rapid-tranquillisation administration | | 29 | 6 | 6.1 | 0.0 | 10.3 | 89.7 | 29 | 6 | 6.1 | 3.4 | 96.6 | 86.2 |  |  |
| 40 | Acute renal impairment | | 29 | 6 | 6.4 | 0.0 | 3.4 | 96.6 | 29 | 6 | 6.1 | 3.4 | 96.6 | 89.7 |  |  |
| 41 | Chronic hepatic impairment | | 29 | 6 | 5.9 | 0.0 | 20.7 | 79.3 | 29 | 6 | 5.8 | 3.4 | 96.6 | 72.4 |  |  |
| 42 | Patients receiving electro-convulsive therapy ^c^ | | 29 | 6 | 5.9 | 0.0 | 20.7 | 79.3 | 29 | 6 | 5.9 | 3.4 | 96.6 | 79.3 |  |  |
| 43 | Patients with non-adherence | | 29 | 6 | 5.7 | 0.0 | 37.9 | 62.1 | 29 | 6 | 5.7 | 3.4 | 96.6 | 65.5 |  |  |
| 44 | Electrolytes levels outside reference range | | 29 | 6 | 5.7 | 0.0 | 37.9 | 62.1 | 29 | 5 | 5.3 | 3.4 | 96.6 | 34.5 |  |  |
| 45 | Patient not reviewed within the past 7 days (acute) or fortnight (rehab) by a pharmacist | | 29 | 5 | 5.3 | 0.0 | 58.6 | 41.4 | 29 | 6 | 5.7 | 3.4 | 96.6 | 62.1 |  |  |
| 46 | Polypharmacy ≥ 10 regular medications | | 29 | 6 | 6.1 | 0.0 | 10.3 | 89.7 | 29 | 6 | 6.0 | 3.4 | 96.6 | 79.3 |  |  |
| 47 | New T2/T3 under the Mental Health Act | | 29 | 5 | 5.1 | 3.4 | 75.9 | 20.7 | 29 | 5 | 5.5 | 3.4 | 96.6 | 48.3 |  |  |
| 48 | Patients with swallowing difficulties/ Nil by mouth | | 28 | 6 | 5.9 | 3.6 | 7.1 | 89.3 | 28 | 6 | 5.8 | 3.6 | 96.4 | 78.6 |  |  |
| 49 | Acute hepatic impairment (Liver function tests > 3 times upper of limit normal) | | 27 | 6 | 6.3 | 0.0 | 3.7 | 96.3 | 27 | 6 | 6.1 | 3.7 | 96.3 | 92.6 |  |  |
| 50 | Medications for Parkinson's disease (e.g. levodopa, apomorphine) | | 29 | 6 | 6.1 | 0.0 | 17.2 | 82.8 | 29 | 6 | 5.8 | 6.9 | 93.1 | 69.0 |  |  |
| 51 | Prescribed direct oral anticoagulant (DOAC) medication | | 29 | 6 | 6.0 | 0.0 | 13.8 | 86.2 | 29 | 6 | 5.7 | 6.9 | 93.1 | 79.3 |  |  |
| 52 | Low sodium levels in a patient taking one or more antidepressants | | 29 | 6 | 5.8 | 0.0 | 37.9 | 62.1 | 29 | 6 | 5.7 | 6.9 | 93.1 | 65.5 |  |  |
| 53 | Increase of a regular psychotropic within 7 days of the last increase (unless as part of a dose titration regimen) | | 29 | 5 | 5.3 | 0.0 | 58.6 | 41.4 | 29 | 5 | 5.4 | 6.9 | 93.1 | 41.4 |  |  |
| 54 | Patient requires oral ‘when required’ psychotropic for agitation | | 29 | 6 | 5.5 | 0.0 | 37.9 | 62.1 | 29 | 6 | 5.6 | 6.9 | 93.1 | 62.1 |  |  |
| 55 | Patients recently moved from another country (difficult to obtain history, different medications brands) | | 29 | 5 | 5.5 | 0.0 | 51.7 | 48.3 | 29 | 6 | 5.4 | 6.9 | 93.1 | 51.7 |  |  |
| 56 | Patients lacking capacity to consent to medication administration | | 29 | 5 | 5.1 | 3.4 | 69.0 | 27.6 | 29 | 5 | 5.2 | 6.9 | 93.1 | 24.1 |  |  |
| 57 | Patients > 80 years (age) | | 29 | 6 | 5.7 | 0.0 | 34.5 | 65.5 | 29 | 6 | 5.7 | 6.9 | 93.1 | 62.1 |  |  |
| 58 | White blood cells (WBC) levels outside reference range | | 29 | 6 | 5.7 | 0.0 | 44.8 | 55.2 | 29 | 5 | 5.4 | 6.9 | 93.1 | 41.4 |  |  |
| 59 | Outstanding Electrocardiogram (ECG) | | 29 | 5 | 5.3 | 0.0 | 69.0 | 31.0 | 29 | 5 | 5.3 | 6.9 | 93.1 | 34.5 |  |  |
| 60 | Warfarin | | 28 | 7 | 6.8 | 0.0 | 0.0 | 100 | 28 | 7 | 6.2 | 7.1 | 92.9 | 75.0 |  |  |
| 61 | Intensive Therapeutic Drug Monitoring (TDM) drugs (e.g. phenytoin, carbamazepine) | | 28 | 6 | 6.2 | 0.0 | 7.1 | 92.9 | 28 | 6 | 5.8 | 7.1 | 92.9 | 78.6 |  |  |
| 62 | Fall > 1 in the preceding 3 months | | 28 | 6 | 5.6 | 0.0 | 39.3 | 60.7 | 28 | 5 | 5.3 | 7.1 | 92.9 | 28.6 |  |  |
| 63 | More than one regular antidepressant prescribed | | 29 | 5 | 5.2 | 3.4 | 55.2 | 41.4 | 29 | 5 | 5.4 | 10.3 | 89.7 | 37.9 |  |  |
| 64 | Prescribed a QTc prolonging medication (e.g chlorpromazine, quetiapine, amisulpride) | | 29 | 6 | 5.7 | 0.0 | 24.1 | 75.9 | 29 | 6 | 5.7 | 10.3 | 89.7 | 75.9 |  |  |
| 65 | Patients with substance abuse | | 29 | 5 | 5.6 | 0.0 | 51.7 | 48.3 | 29 | 5 | 5.3 | 10.3 | 89.7 | 31.0 |  |  |
| 66 | Patients with physical healthcare issues requiring follow-up | | 29 | 6 | 5.6 | 0.0 | 34.5 | 65.5 | 29 | 5 | 5.3 | 10.3 | 89.7 | 41.4 |  |  |
| 67 | Patients > 70 years (age) | | 29 | 5 | 5.2 | 0.0 | 75.9 | 24.1 | 29 | 5 | 5.1 | 10.3 | 89.7 | 20.7 |  |  |
| 68 | Patients with undetermined allergy status | | 29 | 6 | 5.6 | 3.4 | 31.0 | 65.5 | 29 | 6 | 5.7 | 10.3 | 89.7 | 69.0 |  |  |
| 69 | Patients planned for discharge/leave | | 29 | 5 | 5.1 | 0.0 | 75.9 | 24.1 | 29 | 6 | 5.5 | 10.3 | 89.7 | 58.6 |  |  |
| 70 | Patient prescribed medication prompting review of T2/T3 under the Mental Health Act | | 29 | 5 | 4.9 | 3.4 | 75.9 | 20.7 | 29 | 6 | 5.4 | 10.3 | 89.7 | 58.6 |  |  |
| 71 | No VTE assessment for those prescribed antipsychotic. | | 28 | 6 | 5.6 | 3.6 | 25.0 | 71.4 | 28 | 5 | 5.3 | 10.7 | 89.3 | 42.9 |  |  |
| 72 | Patients in seclusion | | 28 | 6 | 5.7 | 0.0 | 32.1 | 67.9 | 28 | 6 | 5.5 | 10.7 | 89.3 | 60.7 |  |  |
| 73 | Moderate hepatic impairment (Liver function tests > upper limit of normal (ULN) | | 27 | 6 | 5.7 | 3.7 | 25.9 | 70.4 | 27 | 6 | 5.4 | 11.1 | 88.9 | 59.3 |  |  |
| 74 | Patients <18 years (age) | | 27 | 6 | 5.7 | 0.0 | 44.4 | 55.6 | 27 | 6 | 5.4 | 11.1 | 88.9 | 51.9 |  |  |
| 75 | Patients on the palliative care pathway | | 26 | 6 | 5.8 | 0.0 | 30.8 | 69.2 | 26 | 6 | 5.7 | 11.5 | 88.5 | 69.2 |  |  |
| 76 | Patients <12 years (age) | | 24 | 6 | 6.1 | 0.0 | 16.7 | 83.3 | 24 | 6 | 5.7 | 12.5 | 87.5 | 70.8 |  |  |
| 77 | Alcohol detox medications (Pabrinex and/or Chlordiazepoxide) | | 29 | 6 | 5.7 | 3.4 | 24.1 | 72.4 | 29 | 6 | 5.6 | 13.8 | 86.2 | 69.0 |  |  |
| 78 | More than one anxiolytic prescribed | | 29 | 5 | 5.4 | 0.0 | 58.6 | 41.4 | 29 | 5 | 5.1 | 13.8 | 86.2 | 27.6 |  |  |
| 79 | Patients who self harm or have suicidal thoughts | | 29 | 6 | 5.7 | 0.0 | 41.4 | 58.6 | 29 | 6 | 5.4 | 13.8 | 86.2 | 51.7 |  |  |
| 80 | Polypharmacy ≥ 5 regular medications | | 29 | 6 | 5.6 | 0.0 | 31.0 | 69.0 | 29 | 5 | 5.1 | 13.8 | 86.2 | 31.0 |  |  |
| 81 | Anti-cancer medications (e.g. azathioprine, fluorouracil) | | 27 | 6 | 6.2 | 0.0 | 3.7 | 96.3 | 28 | 6 | 5.3 | 14.3 | 85.7 | 53.6 |  |  |
| 82 | Haemoglobin levels (CRP, HB1) outside reference range | | 27 | 5 | 5.0 | 0.0 | 85.2 | 14.8 | 27 | 5 | 5.0 | 14.8 | 85.2 | 14.8 |  |  |
| 83 | More than two mood stabilisers prescribed | | 29 | 5 | 5.3 | 0.0 | 58.6 | 41.4 | 29 | 6 | 5.4 | 17.2 | 82.8 | 51.7 |  |  |
| 84 | Patients who have recently stopped or started smoking | | 29 | 5 | 5.3 | 0.0 | 58.6 | 41.4 | 29 | 5 | 5.1 | 17.2 | 82.8 | 27.6 |  |  |
| 85 | Patients prescribed off-label medicines | | 29 | 5 | 5.2 | 0.0 | 62.1 | 37.9 | 29 | 5 | 5.0 | 20.7 | 79.3 | 24.1 |  |  |
| 86 | Patients prescribed medicines by homecare | | 22 | 5 | 4.6 | 0.0 | 100.0 | 0.0 | 22 | 5 | 4.7 | 22.7 | 77.3 | 9.1 |  |  |
| 87 | Phenytoin | | 29 | 6 | 5.9 | 0.0 | 31.0 | 69.0 | 29 | 5 | 5.2 | 24.1 | 75.9 | 48.3 |  |  |
| 88 | Chronic kidney Disease Stage > 2 (slightly reduced eGFR of 60 to 89ml/min, with other signs of kidney damage) | | 29 | 5 | 5.1 | 3.4 | 62.1 | 34.5 | 29 | 5 | 4.9 | 24.1 | 75.9 | 24.1 |  |  |
| 89 | Patients prescribed non-formulary medication | | 29 | 5 | 4.9 | 0.0 | 79.3 | 20.7 | 29 | 5 | 4.9 | 24.1 | 75.9 | 24.1 |  |  |
| 90 | Antimuscarinics (e.g. procyclidine, oxybutynin) | | 29 | 5 | 4.7 | 3.4 | 82.8 | 13.8 | 29 | 5 | 4.9 | 27.6 | 72.4 | 17.2 |  |  |
| 91 | Patients taking laxatives/ have constipation | | 29 | 5 | 4.9 | 3.4 | 69.0 | 27.6 | 29 | 5 | 5.1 | 27.6 | 72.4 | 41.4 |  |  |
| 92 | Chronic kidney Disease Stage > 1 (normal eGFR above 90ml/min, but other tests have detected signs of kidney damage) | | 29 | 5 | 4.7 | 10.3 | 72.4 | 17.2 | 29 | 5 | 4.6 | 27.6 | 72.4 | 10.3 |  |  |
| 93 | Medicines compliance aid from community pharmacy e.g dosette, pillpouch | | 29 | 5 | 4.7 | 0.0 | 96.6 | 3.4 | 29 | 5 | 4.7 | 27.6 | 72.4 | 3.4 |  |  |
| 94 | Digoxin | | 28 | 6 | 5.8 | 0.0 | 25.0 | 75.0 | 28 | 5 | 4.7 | 28.6 | 71.4 | 14.3 |  |  |
| 95 | Patients on self-administration | | 29 | 5 | 4.8 | 3.4 | 86.2 | 10.3 | 29 | 5 | 4.8 | 31.0 | 69.0 | 10.3 |  |  |
| 96 | Patients with new compliance aid requested | | 29 | 5 | 4.9 | 0.0 | 79.3 | 20.7 | 29 | 5 | 4.9 | 31.0 | 69.0 | 24.1 |  |  |
| 97 | Antimicrobials or antivirals (e.g. amoxicillin, nystatin) | | 28 | 6 | 5.4 | 0.0 | 42.9 | 57.1 | 28 | 5 | 4.9 | 32.1 | 67.9 | 32.1 |  |  |
| 98 | Patients diagnosed with COVID | | 27 | 5 | 5.2 | 0.0 | 70.4 | 29.6 | 27 | 5 | 4.7 | 33.3 | 66.7 | 11.1 |  |  |
| 99 | Paroxetine | | 29 | 5 | 4.6 | 3.4 | 86.2 | 10.3 | 29 | 5 | 4.4 | 34.5 | 65.5 | 6.9 |  |  |
| 100 | Hospitalisation due to psychiatric condition > 1 in the preceding year | | 29 | 5 | 5.0 | 0.0 | 75.9 | 24.1 | 29 | 5 | 4.7 | 34.5 | 65.5 | 24.1 |  |  |
| 101 | Patient not spoken to by a pharmacy member within the past 7 days (acute) or fortnight (rehab) | | 29 | 5 | 4.6 | 6.9 | 72.4 | 20.7 | 29 | 5 | 4.7 | 34.5 | 65.5 | 17.2 |  |  |
| 102 | Venlafaxine | | 29 | 5 | 4.6 | 0.0 | 86.2 | 13.8 | 29 | 5 | 4.6 | 37.9 | 62.1 | 10.3 |  |  |
| 103 | Short course of Steroids | | 29 | 5 | 5.0 | 3.4 | 62.1 | 34.5 | 29 | 5 | 4.5 | 37.9 | 62.1 | 10.3 |  |  |
| 104 | Contraceptives (e.g. Yasmin, Evra) | | 28 | 4 | 3.8 | 7.1 | 89.3 | 3.6 | 28 | 5 | 4.3 | 42.9 | 57.1 | 10.7 |  |  |
| 105 | Amiodarone | | 28 | 6 | 5.7 | 0.0 | 32.1 | 67.9 | 28 | 5 | 4.4 | 46.4 | 53.6 | 10.7 |  |  |
| 106 | Theophylline | | 27 | 5 | 5.6 | 0.0 | 51.9 | 48.1 | 27 | 4 | 4.4 | 51.9 | 48.1 | 7.4 |  |  |
| 107 | Hydrocortisone tablets (for adrenal insufficiency/Addison's disease) | | 28 | 6 | 5.7 | 0.0 | 32.1 | 67.9 | 28 | 4 | 4.6 | 53.6 | 46.4 | 25.0 |  |  |
| 108 | Patient taking anti-dementia medications | | 29 | 4 | 4.1 | 6.9 | 82.8 | 10.3 | 29 | 4 | 4.2 | 55.2 | 44.8 | 6.9 |  |  |
| 109 | Aminophylline | | 26 | 5 | 5.4 | 0.0 | 53.8 | 46.2 | 26 | 4 | 3.9 | 65.4 | 34.6 | 11.5 |  |  |
| 110 | Patient taking antidepressants | | 29 | 4 | 3.9 | 10.3 | 82.8 | 6.9 | 29 | 4 | 4.1 | 65.5 | 34.5 | 6.9 |  |  |
| 111 | Patient with daily aseptic needs e.g. on Total Parenteral Nutrition, antibiotic infusion | | 21 | 6 | 6.0 | 0.0 | 19.0 | 81.0 | 21 | 4 | 4.0 | 66.7 | 33.3 | 19.0 |  |  |
| 112 | Hospitalisation due to non-psychiatric condition > 1 in the preceding year. | | 28 | 5 | 4.8 | 0.0 | 82.1 | 17.9 | 28 | 4 | 4.3 | 67.9 | 32.1 | 7.1 |  |  |
| 113 | Digoxin, amiodarone loading | | 25 | 6 | 6.3 | 0.0 | 8.0 | 92.0 | 25 | 4 | 3.8 | 68.0 | 32.0 | 16.0 |  |  |
| 114 | Desmopressin (for cranial diabetes insipidus) | | 24 | 5 | 4.8 | 4.2 | 66.7 | 29.2 | 25 | 4 | 4.1 | 68.0 | 32.0 | 20.0 |  |  |
| 115 | Diuretics | | 29 | 5 | 4.6 | 3.4 | 86.2 | 10.3 | 29 | 4 | 4.1 | 69.0 | 31.0 | 10.3 |  |  |
| 116 | Type I, III or IV antiarrhythmics | | 24 | 5 | 5.0 | 0.0 | 75.0 | 25.0 | 24 | 4 | 4.0 | 75.0 | 25.0 | 8.3 |  |  |
| 117 | Magnesium supplements (e.g. magnesium glycinate, magnesium aspartate) | | 28 | 4 | 3.9 | 7.1 | 85.7 | 7.1 | 28 | 4 | 3.7 | 82.1 | 17.9 | 3.6 |  |  |
| 118 | Angiotensin Converting Enzyme Inhibitors (ACEIs) or Angiotensin Receptor Inhibitors (ARBs) | | 29 | 4 | 4.1 | 6.9 | 86.2 | 6.9 | 29 | 4 | 3.8 | 82.8 | 17.2 | 0.0 |  |  |
| 119 | Tretinoin | | 24 | 5 | 5.3 | 0.0 | 62.5 | 37.5 | 24 | 4 | 3.7 | 83.3 | 16.7 | 8.3 |  |  |
| 120 | Diltiazem | | 27 | 5 | 4.6 | 7.4 | 81.5 | 11.1 | 27 | 3 | 3.3 | 92.6 | 7.4 | 0.0 |  |  |
| 121 | Selective Estrogen Receptor Modulators (SERMs) (e.g. tamoxifen, raloxifene) | | 27 | 4 | 3.8 | 7.4 | 92.6 | 0.0 | 27 | 3 | 3.1 | 96.3 | 3.7 | 0.0 |  |  |
| 122 | Hormone Replacement Therapy (HRT) (e.g. Premique, Premarin) | | 29 | 3 | 3.3 | 17.2 | 79.3 | 3.4 | 29 | 3 | 3.1 | 96.6 | 3.4 | 0.0 |  |  |
| 123 | Bisphosphonates (e.g. alendronate, risedronate) | | 28 | 4 | 3.6 | 7.1 | 92.9 | 0.0 | 28 | 3 | 3.3 | 100.0 | 0.0 | 0.0 |  |  |
|  | | | **^#^**Low-risk if >75% rated 1-2, medium-risk if >75% rated 3-5, and high-risk if >75% rated 6-7. **^##^**Included in the tool if >75% rated importance 6-7 or >85% rated importance 5-7. ^a^ An adverse drug reaction occurred that may have contributed to or resulted in patient harm. ^b^ An interaction occurred that either requires action to be taken to avoid harm or may have contributed to or resulted in patient harm. ^c^ Referring to related medication management issues. PEG: percutaneous endoscopic gastrostomy. NG:nasogastric tube. JEJ:jejunostomy tube. | | | | | | | | | | | | | |

**Appendix.6**

**A flowchart illustrating the refinement process for the risk indicators**

**
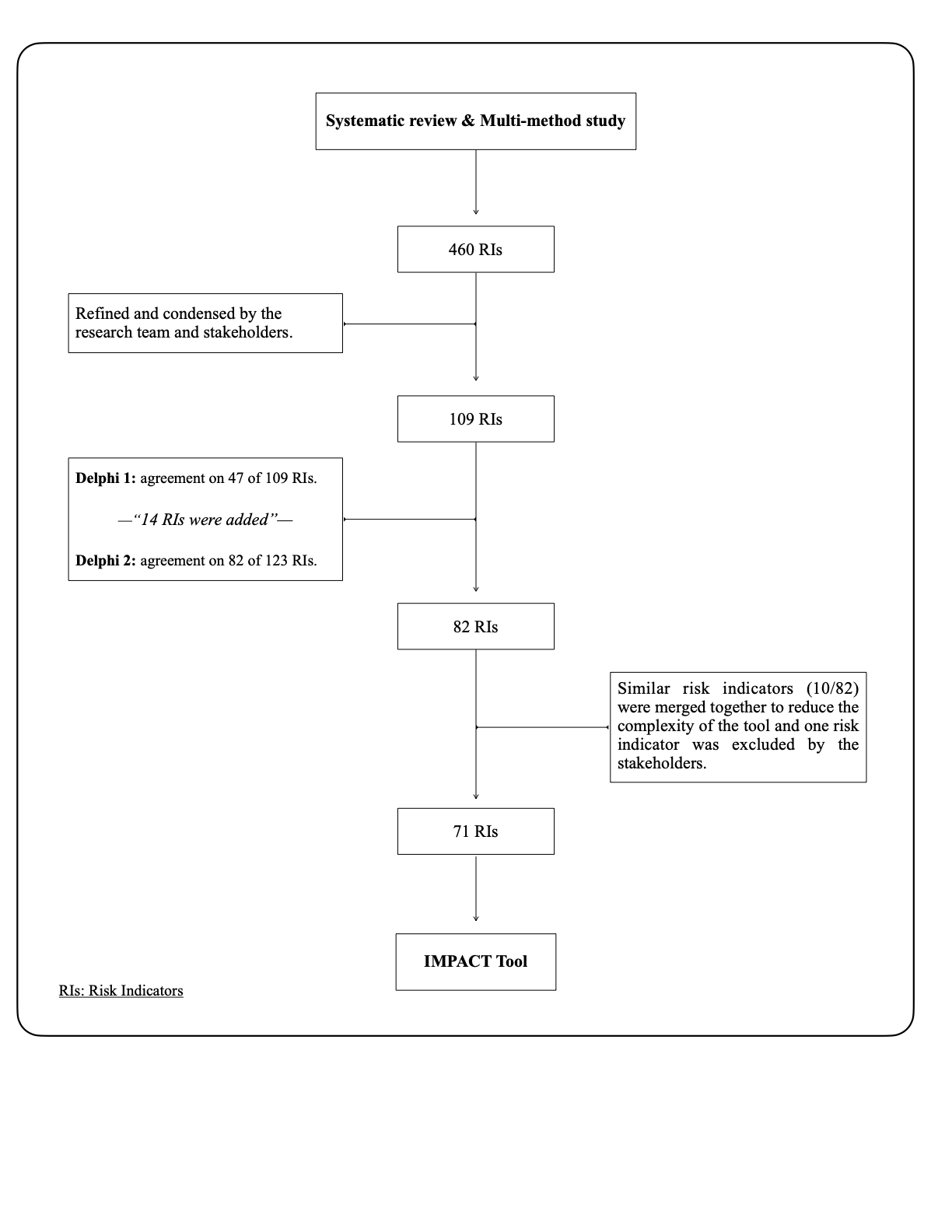
**

**Appendix.7**

**Suggestions for the tool from Delphi study two**

Suggestions included using ‘medicines’ instead of ‘drugs’ in the instruction section, citing the study for the tool development, and fitting the tool in one A4 paper for ease of use. A minority of participants stated that some training would help them understand the tool and how to use it better whereas many believed it was already very clear. All participants preferred an electronic format for the tool such as Excel sheet, a webpage, or integrated into the existing electronic prescribing systems. Few suggested a paper based format mainly so it could be easily stored in the patient file or for emergencies, though few participants were completely against the use of paper format. Last, some concerns were raised about confidentiality and data protection when using webpages or Excel sheets.

**Appendix.8**
